# The Ecological Risks and Invasive Potential of Introduced Ornamental Plants in China

**DOI:** 10.3390/plants14091361

**Published:** 2025-04-30

**Authors:** Haoyu Wang, Min Yang, Xiaohua Ma, Qingdi Hu, Lei Feng, Yaping Hu, Jiehui He, Xule Zhang, Jian Zheng

**Affiliations:** 1 College of Forestry and Biotechnology, Zhejiang A&F University, Hangzhou 311300, China; 2 Key Laboratory of Plant Innovation and Utilization, Institute of Subtropical Crops of Zhejiang Province, Wenzhou 325005, China; yymin0909@163.com (M.Y.);; 3 College of Life and Environmental Science, Wenzhou University, Wenzhou 325035, China

**Keywords:** niche comparison, species distribution model, ornamental plants, alien invasive plants, climate change

## Abstract

The import of ornamental plants has become a major source of alien invasive plants in China, posing threats to local ecosystems. However, research on their invasive potential and management strategies remains limited. This study evaluated the invasion risks of nine representative introduced ornamental plants (including naturalized and invasive species) in China (IOPCs). Using *ecospat* to perform climatic niche comparisons, we found significant unfilling and expansion (>50%) in most introduced ornamental plants, indicating strong invasiveness. Species distribution models (SDMs) were applied to predict the current and future distributions of these IOPCs under four shared socioeconomic pathways (SSPs: SSP1-2.6, SSP2-4.5, SSP3-7.0, and SSP5-8.5) across four time periods (2021–2040, 2041–2060, 2061–2080, and 2081–2100). The SDM results showed that the current high-risk areas are concentrated in southern China. Under future climate change, moderate- and high-risk zones are projected to shift northward, with the total areas increasing significantly, namely moderate-risk areas by 106.10% and high-risk areas by 64.35%, particularly in the border regions of Jiangxi, Fujian, and Zhejiang. We recommend establishing restricted introduction lists for non-native ornamental plants, enhancing monitoring and management in high-risk regions, and implementing early eradication measures. This study quantified the invasion risks and potential distributions of representative invasive ornamental plants, providing a scientific basis for effective control strategies.

## 1. Introduction

The global introduction of ornamental plants has been identified as a major cause of plant invasion [1]. Plant introductions are closely tied to trade intensity [2], and the economic value of ornamental plants may increase invasion opportunities and the associated ecological risks. Many ornamental species have demonstrated invasive potential. For example, *Lantana camara* severely damages native ecosystems through allelopathic effects [3] and is listed among the world’s 100 worst invasive species [3]. *Solidago canadensis*, native to North America [4], was introduced to Europe as an ornamental in the 18th century and became invasive after approximately 100 years [5]. It also exhibits allelopathic properties that inhibit the germination and growth of native species [6]. *Ipomoea cairica*, another invasive ornamental plant in Australia [7], allocates over 46% of its biomass to roots, a strategy that enhances its invasiveness in tropical rainforests [8]. To date, 14,710 alien plant species have been recorded in China, with 933 naturalized and 403 classified as invasive. Among these, the highest proportion originates from the Americas. Hao and Ma found that 72.70% of China’s 403 invasive plants originate from the Americas and 15.38% from Africa [9]. This pattern is attributed to environmental similarities and frequent trade exchanges. Intentional introductions-driven by the perceived value of plants-account for 238 of China’s 403 invasive species, with 139 introduced for ornamental purposes [9]. Invasive ornamental plants are common in China and have been shown to exhibit higher invasiveness than non-ornamental plants, underscoring the urgent need for comprehensive risk assessments of intentionally introduced ornamentals [10]. Although Liu et al. [11] and Hao and Ma [9] have provided detailed descriptions of the species classification, geographical origins, and introduction pathways of alien invasive plants in China, their studies are more focused on summarizing previous works. They have not conducted subsequent invasiveness assessments based on the geographical origin characteristics of alien invasive plants or the fact that ornamental plants are a major source of invasions. It is essential to select appropriate research subjects based on the geographical origin characteristics of alien invasive plants in China, particularly focusing on ornamental plants, which play a significant role in invasions. Assessing their invasiveness and invasion dynamics in China is crucial, yet relevant research remains scarce.

Globalization has accelerated trade and travel, altered species distributions, and broken through historical geographic barriers, exacerbating invasive species challenges [12]. The niche conservatism hypothesis posits that species niches remain stable across time and space [13]. While some studies suggest that invasive species largely conserve their niches in introduced ranges, niche shifts have also been documented [14]. For instance, *Ulex europaeus*, a shrub native to Western Europe, exhibited niche expansions of 49% in Australia, 111% in Northern Europe, 202% in Northwestern America, and 283% in South America [15]. A global analysis of 815 terrestrial plants revealed niche shifts in over 65% of species [16], challenging the niche conservatism hypothesis. To assess niche shifts and quantify invasiveness, multiple metrics (e.g., Schoener’s D [17], Warren’s I [18], and Pianka’s O [19]) have been developed. However, inconsistencies in background selection, spatial resolution, and sampling errors limit their utility. The ecospat package, widely used in invasion biology, offers tools for measuring niche overlap, equivalence testing, and determining dynamic indices, aiding in resolving debates about niche conservatism and informing management strategies [20,21].

Species distribution models (SDMs), grounded in niche conservatism, are widely used to assess invasion risks [22,23]. SDMs can be integrated with GIS to predict potential invasion ranges [24]. Single models like eXtreme Gradient Boosting (XGBOOST) [25] and Random Forest (RF) [26] are commonly employed, but overfitting remains a concern [25]. Ensemble models (e.g., biomod2) combine multiple models for robust predictions [27]. For example, biomod2 was used to evaluate the invasiveness of 11 weed species in China, showing that aggressive climate scenarios may drive the spread of invasive plants northward due to warming [28]. SDMs, when combined with future climate projections, offer insights into dynamic invasion ranges [29]. Comparative studies of current and future distributions, such as for *Spartina alterniflora* in China [30], provide critical data for long-term management.

Introduced ornamental plants in China (IOPCs) are the most significant source of invasive plants within China [9], yet their risks are often underestimated. For instance, *Oxalis debilis*, an IOPC, was studied using Maxent to predict its post-introduction range but only from an invasive species perspective [31]. Although China introduced the Measures for the Management of Invasive Alien Species in 2022, the regulation lacks specific strategies to address the risks posed by ornamental plants [9]. As a result, the invasion potential of IOPCs remains under-recognized in national management efforts. Thus, an IOPC-focused assessment of invasiveness and distribution is urgently needed. This study employed ecospat to evaluate the current invasiveness of nine IOPCs in China, biomod2 to predict their current and future distributions under climate change, and regression analysis to link warming trends with habitat expansion. We propose management strategies to mitigate IOPC risks, contributing to the development of science-based policies.

## 2. Results

### 2.1. Climate Niche Comparison of Native vs. Introduced Ranges in Nine IOPCs

**Figure 1:**
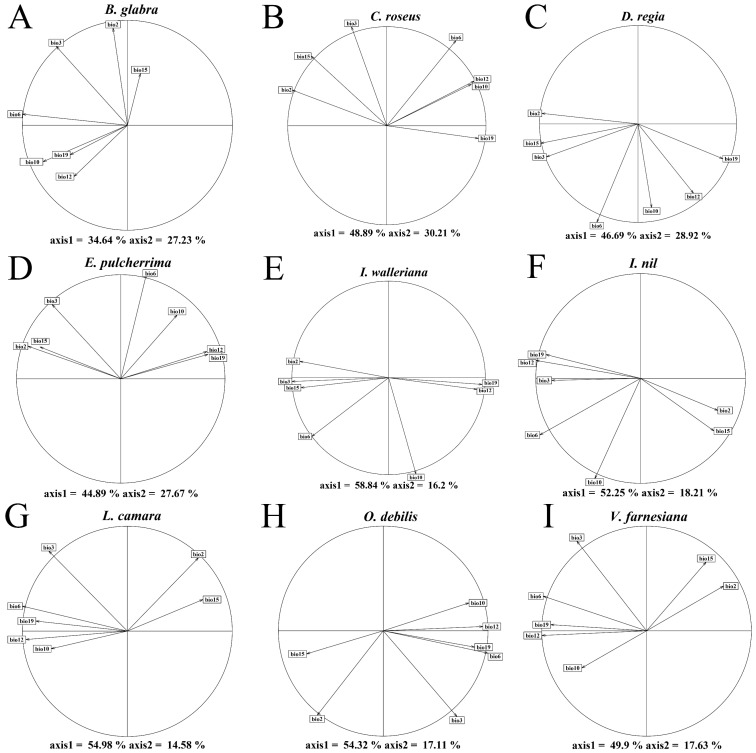
PCA-env-based correlation circle of bioclimatic factors.

**Figure 2:**
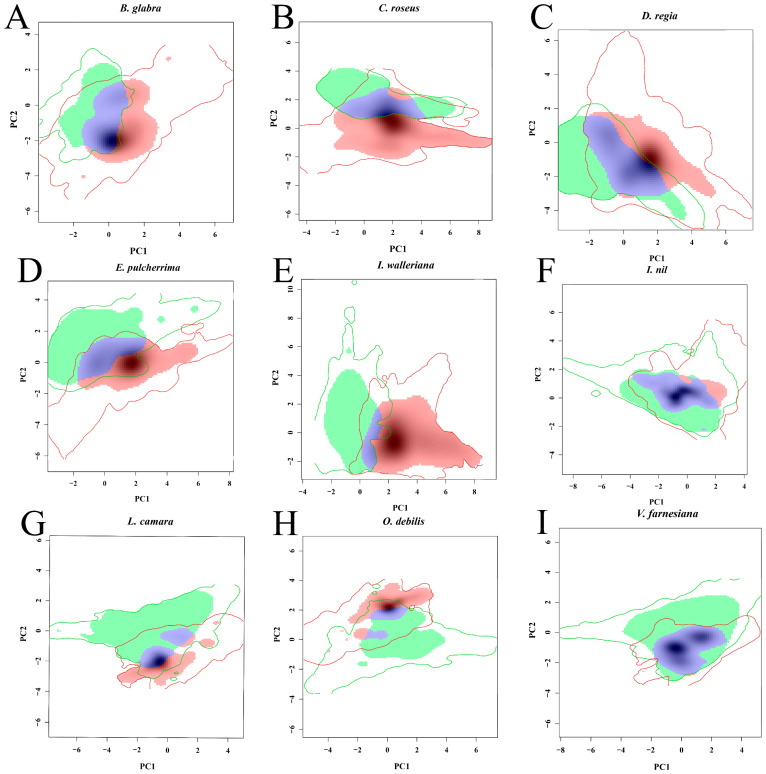
Climatic niche overlap analysis between native and introduced ranges (China) of the nine IOPCs. The red solid line represents the climatic niche range of the introduced range (China), while the green solid line denotes the climatic niche range of the native range. Gray shading indicates the kernel density estimation of the climatic niche in the introduced range (China). Red areas denote niche expansion, blue areas represent niche stability, and green areas indicate unfilling.

The climatic niches of nine introduced ornamental plants (*Bougainvillea glabra*, *Catharanthus roseus*, *Delonix regia*, *Euphorbia pulcherrima*, *Impatiens walleriana*, *Ipomoea nil*, *Lantana camara*, *Oxalis debilis*, and *Vachellia farnesiana*) in their native range and introduced range in China were compared. Principal component analysis (PCA) revealed that the first two principal components explained 60–80% of the variance, with *B. glabra* having the lowest cumulative variance (61.87%) and *C. roseus* the highest (79.1%) (Figure 1). Along the first principal component (PC1), niche differences were observed in *B. glabra*, *D. regia*, and *I. walleriana*, mainly driven by temperature and precipitation variability. Specifically, *B. glabra* was influenced by low temperatures, while *D. regia* and *I. walleriana* were associated with both temperature and precipitation variation. For the second principal component (PC2), *C. roseus*, *E. pulcherrima*, *L. camara*, and *O. debilis* showed distinct niche patterns. These were mainly related to isothermality, temperature variability, and precipitation, with *C. roseus* and *E. pulcherrima* closely associated with isothermality and low-temperature conditions (Figure 1 and Figure 2).

Schoener’s D value ranges from 0 to 1, representing complete non-overlap to complete overlap of climatic niches, indicating the degree of overlap between the native and introduced climatic niches. In this study, the average Schoener’s D for the nine IOPCs was 0.0807. *B. glabra* exhibited the highest Schoener’s D value, while *I. walleriana* had the lowest D value of 0.0069 (Table 1). The majority of IOPCs exhibit expansion values exceeding 50%, indicating higher invasiveness potential. Expansion values range from 0 to 1, with their sum equaling 1, representing the newly occupied portion of the climatic niche in the introduced range. Five IOPCs (*B. glabra*, *C. roseus*, *E. pulcherrima*, *I. walleriana*, and *O. debilis*) exceeded 0.5 in expansion, suggesting that more than half of their climatic niches in China are newly expanded areas. Notably, *I. walleriana* showed the highest expansion (0.9439), with 94.39% of its climatic niche in China being newly occupied. Similarly, the majority of IOPCs exhibited unfilling values exceeding 0.5, indicating significant potential for future invasions. Unfilling refers to the portion of the native climatic range that remains unoccupied in the introduced range. Seven IOPCs (*C. roseus*, *D. regia*, *E. pulcherrima*, *I. walleriana*, *L. camara*, *O. debilis*, and *V. farnesiana*) exceeded 0.5 in unfilling, with *L. camara* showing the highest value (0.8856), meaning that 88.56% of its native climatic niche remains unoccupied in its introduced range in China. Among the nine IOPCs, only *B. glabra* exhibited significant niche similarity (niche similarity test, *p* < 0.05), indicating a statistically significant similarity between its native climatic niche and the climatic niche in China. For the remaining eight IOPCs, the niche similarity test results were not statistically significant (*p* > 0.05, Table 1). In the niche equivalency tests, six IOPCs (*C. roseus*, *D. regia*, *I. walleriana*, *I. nil*, *L. camara*, and *O. debilis*) showed non-significant results (*p* = 1), suggesting that the climatic niches of most IOPCs remain conservative in their introduced ranges. In contrast, three IOPCs (*B. glabra*, *E. pulcherrima*, and *V. farnesiana*) displayed significant results (niche equivalency test, *p* < 0.05), indicating a statistically significant divergence between their native climatic niches and those in China (Table 1).

### 2.2. Changes in Potentially Suitable Areas Under Current Climate Conditions

**Figure 3:**
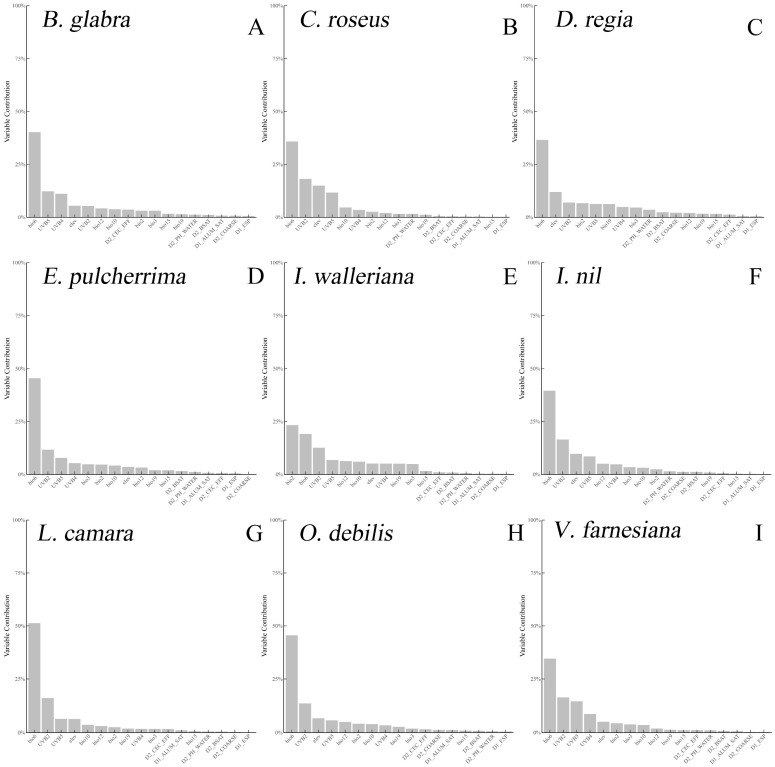
Contribution rates of environmental variables to the species distribution models (SDMs) for the nine IOPCs.

Although SDMs are constructed using multiple environmental variables, their contributions to the models are not uniform and are typically quantified with varying importance or contribution rates. Among the variables in the models for the nine IOPCs, bio6 (minimum temperature of the coldest month) ranked first in contribution for eight IOPCs, except for *I. walleriana*, where bio6 ranked second (Figure 3). Bio6 had relatively high contributions across the SDMs for all nine IOPCs, suggesting that extreme low temperatures in winter may significantly influence their adaptation and distribution in China.

**Figure 4:**
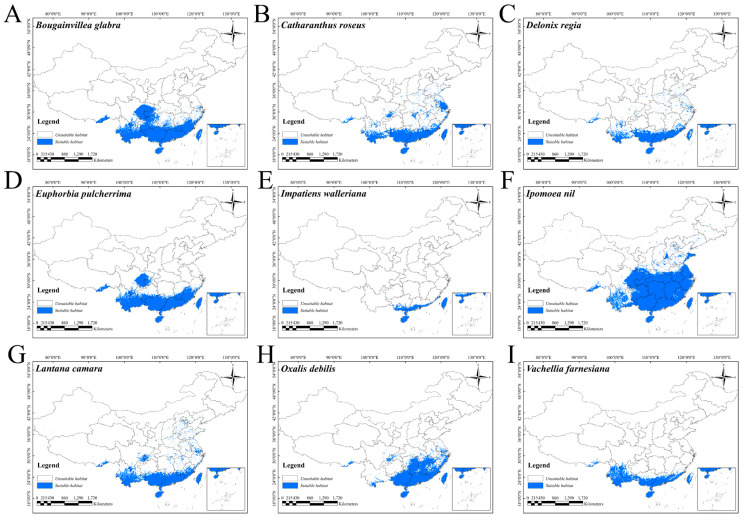
Current potentially suitable areas for the nine IOPCs in China. Blue represents potentially suitable areas. Blank areas indicate non-potentially suitable regions.

Among these nine IOPCs, *I. nil* had the largest potential distribution area, covering 215.60 × 10^6^ km^2^, whereas *I. walleriana* had the smallest area (18.08 × 10^6^ km^2^) (Figure 4 and Figure S1). The potential distribution areas of the remaining seven IOPCs fell between these two extremes (Figure S1).

### 2.3. Predicted Distribution Dynamics of IOPCs Under Climate Change

**Figure 5:**
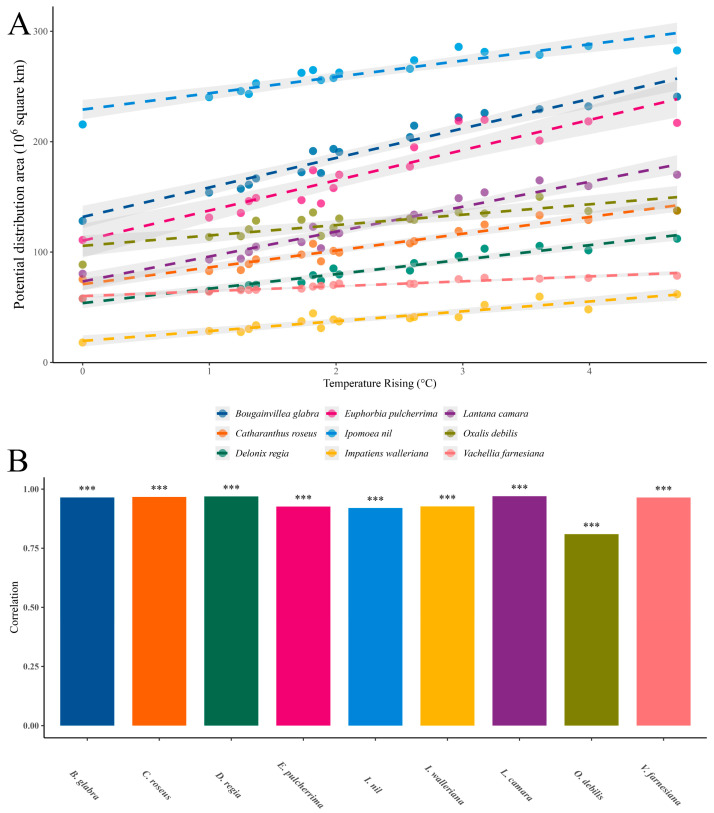
Regression analysis of temperature rise and potentially suitable areas for IOPCs under future climate conditions: (**A**) regression analysis with 95% confidence intervals; (**B**) correlation and significance tests. *** indicates *p* < 0.001, signifying extremely significant.

Regression analysis was employed to identify relationships between the variables. To investigate the potential association between climate warming and the future distribution of IOPCs, univariate regression analyses were conducted between temperature rise and the projected suitable habitat area for each IOPC (Figure 5, Table 2 and Table 3). Notably, climate warming showed a significant positive correlation with the potentially suitable area of all nine IOPCs (*p* < 0.001, Figure 5), indicating that the future distributions of these IOPCs may be strongly influenced by warming. The R^2^ value ranges from 0 to 1, with no absolute threshold for quality. However, an R^2^ > 0.8 is generally considered indicative of good model fit, while an R^2^ between 0.5 and 0.8 indicates moderate explanatory power. The average R^2^ across the SDMs for the nine IOPCs was 0.87, demonstrating relatively reliable regression results. The mean regression coefficient was 15.85 × 10^6^ km^2^/°C, implying that a 1 °C temperature increase would expand the average potential distribution area of the nine IOPCs by 15.85 × 10^6^ km^2^. *B. glabra* exhibited the highest regression coefficient (26.72 × 10^6^ km^2^/°C), while *V. farnesiana* had the lowest (4.47 × 10^6^ km^2^/°C) (Table 2).

**Figure 6:**
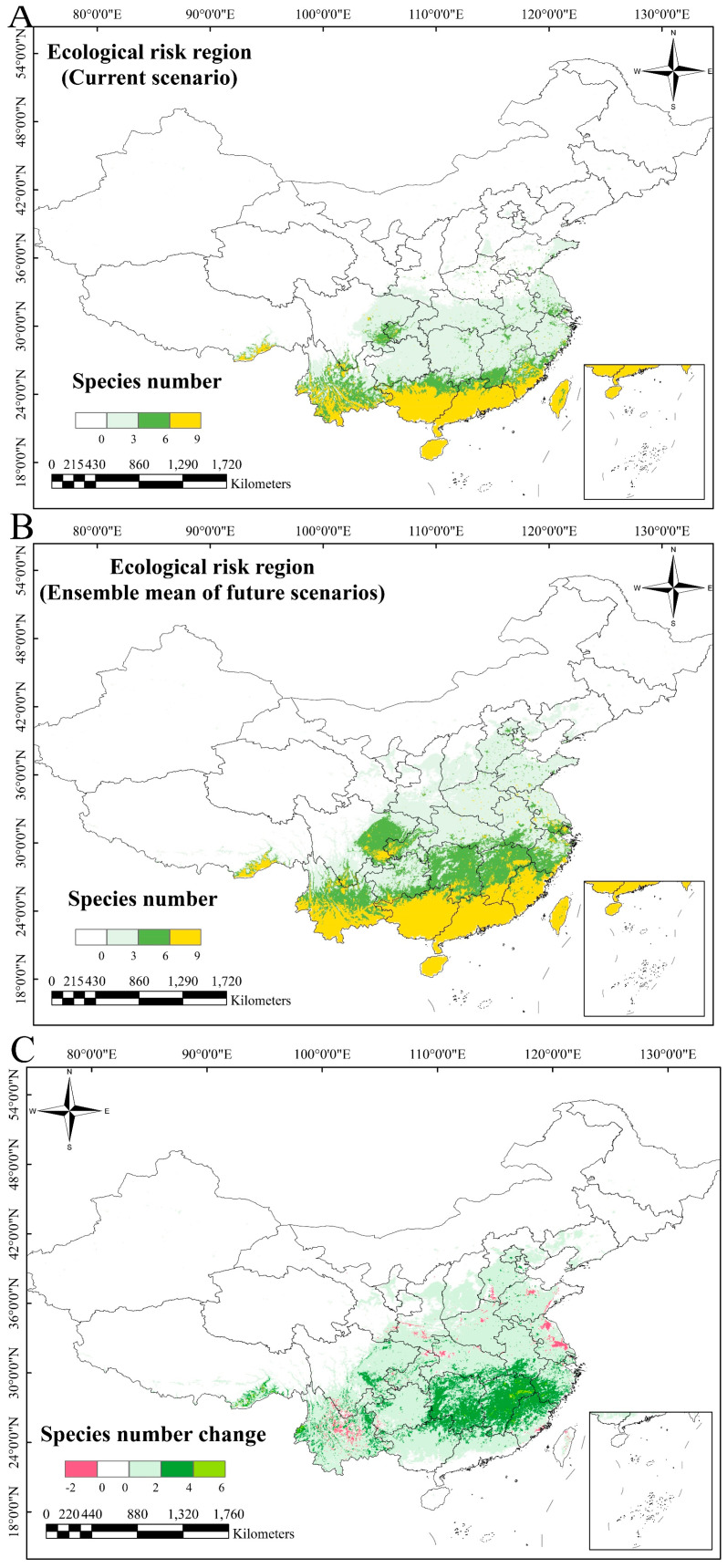
Ecological risk zones and risk dynamics for IOPCs under climate change: (**A**) current ecological risk zones for IOPCs; (**B**) mean ecological risk zones for IOPCs under future scenarios; (**C**) ecological risk dynamics of IOPCs under climate change scenarios.

The ecological risk zones were classified into four levels based on the number of IOPCs with potential distributions in each region under current and future scenarios: low-risk regions (0–3 species), moderate-risk regions (3–6 species), high-risk regions (6–9 species), and unsuitable regions (no species). Under the current conditions, high-risk zones cover 39.06 × 10^6^ km^2^, moderate-risk zones cover 50.35 × 10^6^ km^2^, and low-risk zones cover 140.76 × 10^6^ km^2^ (Table 2). The current high-risk areas are concentrated in Guangxi, central and southern Guangdong, southern Fujian, the low- and moderate-altitude regions of Yunnan and Taiwan, and Hainan. The moderate-risk zones surround the northern edges of the high-risk zones, while the low-risk zones border the northern peripheries of the moderate-risk zones. The risk gradient decreased from southern to higher-latitude northern areas (Figure 6A).

In future scenarios, the high-risk areas will expand to 82.75 × 10^6^ km^2^, the moderate-risk areas will expand to 80.50 × 10^6^ km^2^, and the low-risk areas will expand to 152.17 × 10^6^ km^2^. The northward decline in risk levels will persist (Figure 6B), but all risk zones will increase in size compared to the current conditions (Table 2).

The comparative analysis of the current and future distributions revealed dynamic shifts under climate change. The future scenarios showed substantial increases in moderate- and high-risk areas. Currently, low-risk zones are 57.43% larger than the combined area of moderate- and high-risk zones. However, under future scenarios, low-risk zones will become 6.79% smaller than the combined moderate- and high-risk areas. While low-risk zones will increase by 11.41%, moderate- and high-risk zones will expand by 106.10% and 64.35%, respectively (Table 2). Climate change-driven increases in the moderate- and high-risk zones will be most pronounced in Hunan, Jiangxi, northern Fujian, and southern Zhejiang (Figure 6C). The border regions of eastern Jiangxi, southwestern Zhejiang, and northern Fujian will experience the most dramatic species count changes, whereas southern Guangxi, Guangdong, Hainan, and Taiwan will maintain relatively stable species numbers (Figure 6C).

## 3. Discussion

This study was based on the fact that invasive alien plants in China predominantly originate from the Americas [9]. Among the nine selected IOPCs, six (67%) were from the Americas, aligning with previous findings that 70% of China’s invasive plants originate from this region [9]. To enhance generalizability, three additional IOPCs from Africa were included. However, the conclusions should be interpreted with caution, as the limited number of species restricts broader applicability. By integrating niche comparisons [15] and SDMs [32], we comprehensively assessed the ecological risks and potential invasion ranges of these IOPCs under current and future climate scenarios. Future studies should expand the taxonomic breadth of the niche comparisons to derive more generalized insights into ornamental plant invasiveness in China.

### 3.1. Niche Comparison of the Nine IOPCs

Only *B. glabra* showed significant niche similarity (*p*< 0.05). Biogeographic processes (e.g., dispersal or adaptation) can indirectly drive niche shifts by altering the environmental conditions [33]. It is important to note that the results of niche tests are based on statistical inference. Therefore, a non-significant result in a niche similarity test only indicates that ecological similarity between the compared areas cannot be statistically confirmed. Niche equivalency tests showed non-significant results (*p* = 1) for six IOPCs (*C. roseus*, *D. regia*, *I. walleriana*, *I. nil*, *L. camara*, and *O. debilis*), indicating niche conservatism in their introduced ranges. These results are consistent with studies comparing North American-East Asian congeners, which suggest that niche conservatism dominates, while non-conservatism (*p* < 0.05) may arise from environmental disparities [34]. While the niche conservatism hypothesis has been challenged in recent years, the findings of this study provide a degree of support for its validity [35].

Notably, five IOPCs (*B. glabra*, *C. roseus*, *E. pulcherrima*, *I. walleriana*, and *O. debilis*) showed an expansion value of >50%, indicating that over half of their climatic niches in China are newly occupied [36]. The high unfilling (>50%) for seven IOPCs (*C. roseus*, *D. regia*, *E. pulcherrima*, *I. walleriana*, *L. camara*, *O. debilis*, and *V. farnesiana*) suggests untapped invasion potential [37]. The invasion of alien species has been demonstrated to be closely linked to their niche expansion in new environments, often leading to the extinction of native species or increased competition [38]. *L. camara* has shown significant niche expansion in India, with an expansion value of 0.20 and an unfilling value of 0.23 [39]. However, in this study, its expansion in China exceeded 0.40, and its unfilling surpassed 0.80. This suggests that IOPCs may exhibit stronger invasiveness and greater potential for future invasion in China. Methodological limitations, such as Schoener’s D sensitivity to the spatial resolution and sampling density, highlight the need for multi-scale assessments. Broennimann et al. mitigated sampling bias via kernel density estimation, but resolution-dependent Schoener’s D reductions persisted [40].

### 3.2. Implications of IOPC Potentially Suitable Area Predictions

SDMs may not fully match actual species ranges due to imperfect detection and reliance on limited ecological data [41]. The accuracy of models depends on data quality. Spatial autocorrelation from clustered occurrences [42,43] and multicollinearity between variables [44] can introduce bias. Data sparsification reduces autocorrelation but may reduce model performance, especially for rare species [45]. To improve predictions, we removed highly correlated variables based on relevance and thinned occurrence points according to environmental resolution. Model accuracy was assessed using the true skill statistic (TSS) and receiver operating characteristic (ROC) [46]. TSS supports threshold-based classification but is sensitive to threshold selection and can affect spatial outputs [47]. ROC is independent of threshold but can be inflated by pseudo-absence points [48]. A combined evaluation using TSS and ROC was used to ensure robustness. The final ensemble models for the nine IOPCs achieved strong performance (TSS > 0.7, ROC > 0.95).

The regression analysis revealed a strongly positive correlation between warming and habitat expansion (*p* < 0.001). However, bio6 (minimum winter temperature) was the predominant variable in the SDMs, suggesting that extreme cold relaxation drives distribution shifts. Future studies should incorporate phenotypic plasticity under low temperatures [49] and nonlinear responses (e.g., lagged effects) using SEMs or GAMs [50]. All nine IOPCs were predicted to expand their habitats northward in the future. The northward expansion of invasive alien plant species in China under climate change scenarios has been confirmed by several studies. For four invasive alien plants in China (*Ageratina adenophora*, *Alternanthera philoxeroides*, *Ambrosia artemisiifolia*, and *Mikania micrantha*), their potentially suitable habitats under climate change scenarios also show a northward shift [51]. For invasive alien weeds in China, the increase in future potential suitable habitat areas was significantly positively correlated with an increase in latitude [52]. In this study, regression analysis indicated a significant positive correlation between climate warming and an increase in potential suitable habitat areas of IOPCs. Given the high contribution of the bio6 factor in the SDMs of IOPCs, it is speculated that the weakening of extreme low winter temperatures may be a key factor driving the northward shift of IOPCs in regions at moderate and high ecological risk. Discrepancies among GCMs (e.g., HadGEM, MIROC, and CCSM) in physical assumptions and atmospheric processes [53], as well as scenario differences (RCP vs. SSP), may explain the variability. The BCC-CSM2-MR model was selected for its superior performance in East Asia’s monsoon climate [54]. Despite the projected warming (up to 4.69 °C for IOPCs vs. 6.87 °C for fast-growing trees [55]), the IOPCs’ thermal tolerance thresholds may not be exceeded, enabling unified expansion trends.

### 3.3. Ecological Risk Management

The niche comparisons and SDMs highlight persistent invasion risks, particularly in southern China (Yunnan, Guangxi, Guangdong, Hainan, and Taiwan), aligning with the documented high invasion frequencies. Given that 58.40% of invasive plants were intentionally introduced as ornamentals [9], and invasiveness often exhibits lag effects [56], stricter regulations are critical. We propose a dual “whitelist–blacklist” system for border regions. The blacklist will restrict the entry of species post-invasion, though ecological damage may have already occurred [57]. The whitelist will only permit the entry of proven non-invasive species, which will require rigorous risk assessments [58].

Future high-risk zones (e.g., the Jiangxi–Fujian–Zhejiang border) require intensified monitoring efforts and real-time data sharing. Early detection and eradication will minimize costs [59], while containment (e.g., buffer zones) and mitigation (e.g., native vegetation restoration [60,61]) can curb the spread of established invasions.

## 4. Materials and Methods

### 4.1. Occurrence Points of IOPCs

Over 50% of invasive plants and 85% of invasive woody species were initially introduced for ornamental and landscaping purposes [62]. However, research on the invasiveness of ornamental plants using niche comparisons and SDMs remains limited. Therefore, in this study, we selected nine representative ornamental plants that are widely distributed in China from the China List of Alien Invasive and Naturalized Plants (2023) (https://www.cvh.ac.cn/iapc/ (accessed on 3 December 2024)).

The selection criteria were as follows: the species must be an ornamental plant; the species must have documented distributions in both China and its native range to facilitate the analyses; and the species must meet acceptable thresholds for SDM accuracy. The final selection included nine species: *B. glabra*, *C. roseus*, *D. regia*, *E. pulcherrima*, *I. walleriana*, *I. nil*, *L. camara*, *O. debilis*, and *V. farnesiana*. Occurrence data for these plants in China and their native ranges were obtained from the Global Biodiversity Information Facility (GBIF, https://www.gbif.org/ (accessed on 6 December 2024)), which were supplemented with records from the Chinese Virtual Herbarium (CVH, https://www.cvh.ac.cn/ (accessed on 16 December 2024)). To ensure credible and traceable native ranges for the nine IOPCs introduced to China, Plants of the World Online (POWO, https://powo.science.kew.org/ (accessed on 17 January 2025)) served as the unified reference.

**Figure 7:**
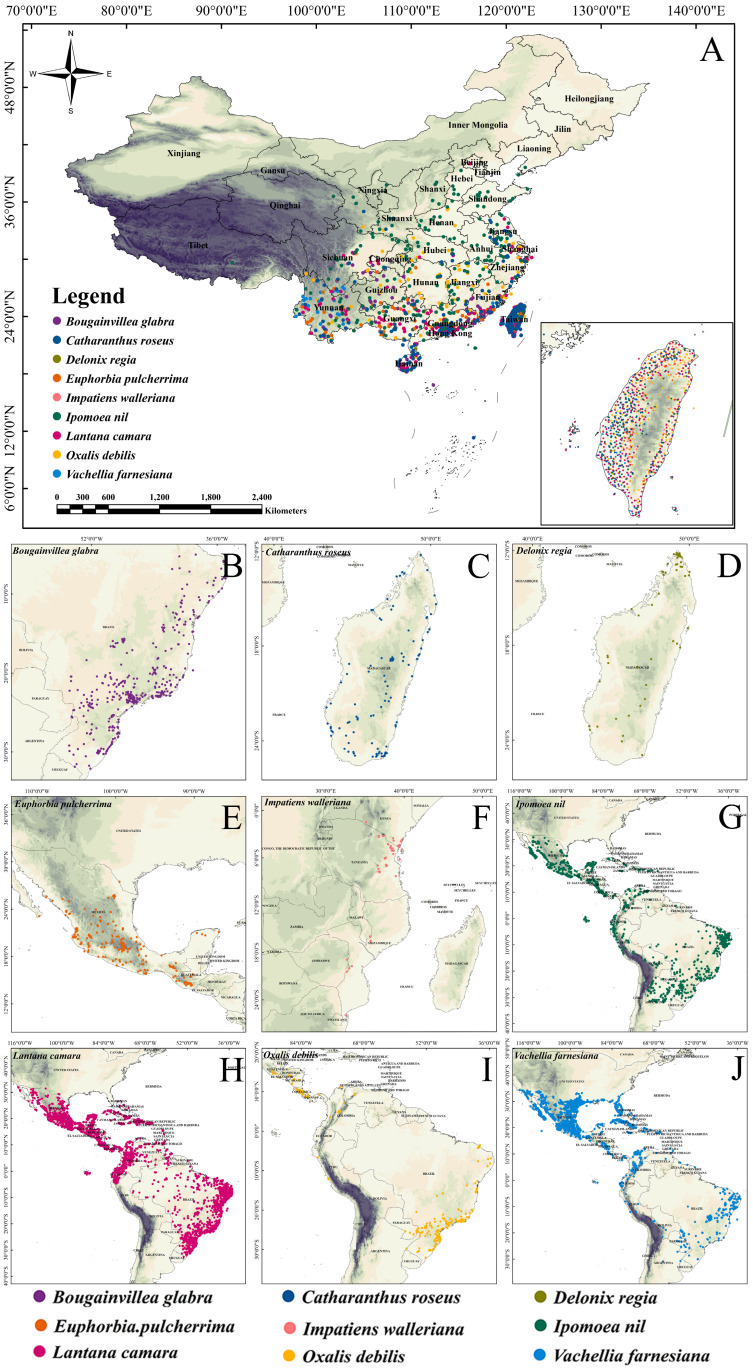
Geographic distribution of occurrence points of IOPCs in (**A**) China and (**B**–**J**) their native ranges.

Data from the GBIF and CVH could not be directly used for modeling. Many records lacked precise coordinates, and both databases contained duplicate entries (i.e., repeated records for the same location at the same or different times). In addition, GBIF distribution data are primarily classified by administrative units, whereas the native range of a plant species typically does not follow political boundaries, making it difficult to accurately define native status at the country level. To improve the data quality, the following cleaning steps were applied to the occurrence points of the nine IOPCs in China and their native ranges: the filtering of native range records based on the POWO database, retaining only those occurrences that fall within the documented native range of each species; the removal of data points without accurate coordinates and duplicates with identical coordinates; the exclusion of uncertain outliers where the environmental conditions exceeded the species’ known ecological tolerance thresholds; and spatial thinning to ensure that no more than one occurrence point per 2.5′ (5 km × 5 km) grid cell, minimizing spatial autocorrelation effects. After processing, 2174 occurrence points in China (Figure 7A) and 7558 native range points (Figure 7B–J) were retained for the nine IOPCs.

### 4.2. Environmental Variables

The bioclimatic variables for the current and future scenarios were downloaded from WorldClim (https://worldclim.org/ (accessed on 3 January 2025)). The future climate projections were based on the BCC-CSM2-MR model [54], which performs well in simulating East Asian climates. BCC-CSM2-MR emphasizes the impact of extreme high temperatures, which may align more closely with the “continental” characteristics of the East Asian monsoon climate (e.g., high summer temperatures and large seasonal temperature variation) [63]. In the future predictions of suitable habitats for *Pseudoechthistatus*, the BCC-CSM2-MR model projects a reduction in suitable areas, while the MIROC6 model predicts an increase in suitable areas [63]. Four shared socioeconomic pathways (SSPs)—SSP1-2.6 (low radiative forcing), SSP2-4.5 (moderate radiative forcing), SSP3-7.0 (moderate–high radiative forcing), and SSP5-8.5 (high radiative forcing)—were selected, each spanning four periods: 2021–2040, 2041–2060, 2061–2080, and 2081–2100. Ultraviolet radiation data were obtained from the Global UV-B Radiation Dataset (https://www.ufz.de/gluv/ (accessed on 6 January 2025)), and soil variables (HWSD2.0) were sourced from the Harmonized World Soil Database (https://gaez.fao.org/pages/hwsd (accessed on 7 January 2025)).

HWSD2.0 divides soil factors into seven depth layers. Surface soil layers D1 (0–20 cm) and D2 (20–40 cm), which most strongly influence plant roots and have optimal data coverage [64], were selected as variables for the SDMs. Soil and UV-B variables were resampled to 2.5′ to match the bioclimatic and elevation data from WorldClim (2.5′ resolution). To mitigate multicollinearity, variables with correlation coefficients |*r*| > 0.8 were iteratively excluded based on the contribution rankings [65]. Bio10 (mean temperature of the warmest quarter) was retained due to its relevance to climate warming impacts on invasive plants [28] despite its strong negative correlation with elevation (*r* = −0.94; Figure S2). Seventeen environmental variables were ultimately used for SDM construction (Table S1).

### 4.3. Construction of the Species Distribution Model

SDMs were developed using the *biomod2* package [66] in R (v4.1.3), following the official documentation (https://biomodhub.github.io/biomod2/reference/index.html (accessed on 10 March 2025)). For each species, 1000 pseudo-absence points were generated three times to ensure reliability [67]. Ten algorithms were employed: Artificial Neural Network (ANN), Classification Tree Analysis (CTA), Flexible Discriminant Analysis (FDA), Generalized Additive Model (GAM), Generalized Boosting Model (GBM), Generalized Linear Model (GLM), Multivariate Adaptive Regression Splines (MARS), Maximum Entropy (MAXNET), RF, and XGBOOST. The occurrence data were split into training (80%) and validation (20%) sets. Model accuracy was evaluated using TSS and ROC.

Single models with a TSS > 0.7 were integrated into ensemble models via the EMwmean (weighted mean ensemble) method [68], prioritizing higher-accuracy models. The final SDMs predicted the current (Figure 6) and future (Figures S9–S17) potentially suitable areas for the nine IOPCs.

(1) $$SDM=\frac{\sum_{i=1}^{n}w_{i}P_{i}}{\sum_{i=1}^{n}w_{i}}$$

*w_i_*: The weight of the *i*th model;
*P_i_*: The prediction probability of the *i*th model;
*n*: The number of individual models used to construct the final ensemble model.

### 4.4. Model Accuracy Evaluation

In this study, TSS and ROC curve were used as criteria to evaluate model accuracy. Specifically, models with TSS values of ≥0.6 and ROC values of >0.85 were considered to have high reliability. For the nine IOPCs in this study, most single models met these reliability standards (Figure S3). However, the underperformance of some single models for specific IOPCs was inevitable. For example, the XGBOOST model for *B. glabra* had an average TSS of only 0.617. Despite this, the mean TSS and ROC values for all IOPC models met the reliability requirements.

To further improve model accuracy, we used the EMwmean method to integrate single models into ensemble models for each IOPC. The results of the integrated models showed that, except for *Ipomoea nil* (TSS = 0.746), the TSS values for the SDMs of the remaining eight IOPCs exceeded 0.8, and all IOPC models achieved ROC values greater than 0.95. This indicates that the SDMs for the nine IOPCs in this study exhibited exceptionally high accuracy and reliability (Figure S4).

### 4.5. Niche Comparisons: Niche Overlap and Niche Tests

The occurrence points for the nine IOPCs in their native range and introduced range in China were spatially thinned to reduce the impact of spatial autocorrelation on the analyses. The environmental background for the niche comparisons comprised seven bioclimatic variables (bio2, bio3, bio6, bio10, bio12, bio15, and bio19). The definition of the environmental backgrounds directly influences niche comparison outcomes [20], and expanding background regions may reduce Schoener’s D [69]. Thus, the background extents were uniformly defined by the geographic distribution of the IOPC occurrence points. Minimum convex polygons (MCPs) were generated using the mcp function in the adehabitatHR package (v0.4.22) [70] to delineate environmental backgrounds.

Principal component analysis (PCA-env) was performed using the dudi.pca function in the ade4 package (v1.7.22) [40] to quantify the climatic niches. Biplots (Figure 2) were used to visualize the contributions of the bioclimatic factors to the principal components. Niche quantification based on the PCA scores enabled the subsequent niche comparisons (Figure S5 and S6).

Schoener’s D, ranging from 0 (no overlap) to 1 (complete overlap) [71], was calculated via the ecospat.niche.overlap function in the ecospat package (v3.5). Niche dynamics were assessed using three indices: unfilling, which is the proportion of the native niche that is unoccupied to that in the introduced range (0 = fully filled; 1 = fully unfilled), indicating invasion potential [71]; stability, which indicates the overlap between native and introduced niches, reflecting conservatism [72]; and expansion, which is the ratio between the size of the introduced niche to that of the native niche, indicating adaptability [72]. The sum of the stability and expansion values equals 1.

Niche similarity and equivalency tests were conducted using ecospat.niche.similarity.test and ecospat.niche.equivalency.test [18] (Figures S7 and S8). The similarity test evaluates whether the niche overlap exceeds random expectations (null hypothesis: overlap = random). Significant results (*p* < 0.05) indicate that the null hypothesis should be rejected and suggest niche similarity. The equivalency test assesses niche identity (null hypothesis: niches are identical). Significant results (*p* < 0.05) indicate niche divergence. Both tests were replicated sufficiently to ensure reliability [18].

### 4.6. Regression Analysis Under Climate Change

Following Zhao et al. [55], regression analysis was used to explore the relationships between warming and IOPC habitat expansion. Current potentially suitable areas for all nine IOPCs were overlaid, and the mean annual temperature (bio1) of the overlapping region was calculated to quantify the warming intensity. Univariate regression between temperature rise and habitat area was performed using the lm, cor, and cor.test functions in R’s stats package. The results were visualized using ggplot2 (R v4.1.3).

### 4.7. Classification of Ecological Risk Zones

The ecological risk levels were classified based on the number of IOPCs per grid cell (low-risk region: 0–3 species; moderate-risk region: 3–6 species; high-risk region: 6–9 species; and unsuitable region: 0 species). Sixteen future scenarios (4 SSPs × 4 periods) were averaged to predict the risk dynamics. Changes in species counts were categorized as follows: risk reduction for -2 to 0 species; mild risk increase for 0–2 species; moderate risk increase for 2–4 species; high risk increase for 4–6 species; and no change for 0 species (excluding unsuitable regions)

## 5. Conclusions

This study, through niche comparisons and SDMs, revealed the invasion risks and invasive potential of nine representative introduced ornamental plants in China. The niche analysis demonstrated that most of these IOPCs exhibited unfilling and expansion exceeding 50%, indicating significant niche vacancy and expansion, which suggests strong invasive potential. The SDM predictions highlighted that current high-risk areas are primarily concentrated in southern China. Under future climate change scenarios, moderate- and high-risk zones are projected to expand significantly northward, with the most dramatic ecological risk increases observed in the border regions of Jiangxi, Fujian, and Zhejiang. The results quantified the invasion risks of introduced ornamental plants in China and predicted their potential distributions under varying climate scenarios, providing a scientific basis for adaptive management strategies. Based on these findings, we recommend implementing periodic reassessments of high-risk regions and integrating these insights into China’s 2022 invasive species management measures. This includes imposing restrictions on the introduction of non-native ornamental plants, enhancing monitoring of high-risk areas, and prioritizing early eradication efforts. While this study offers critical insights that can aid us in understanding and managing the invasion risks of introduced ornamental plants, future research should incorporate a broader range of species to comprehensively assess their invasive potential under climate change.

## Figures and Tables

**Table 1:** Overlap indices, dynamic indices, and equivalency/similarity test results for climatic niche comparisons (native distribution range vs. introduced distribution range in China) of the nine IOPCs.

IOPC	Niche Overlap (D)	Niche Similarity (*p*)	Niche Equivalency (*p*)	Unfilling	Stability	Expansion
*Bougainvillea glabra*	0.2153	0.0350	0.0099	0.3277	0.4880	0.5120
*Catharanthus roseus*	0.0665	0.1538	1.0000	0.6758	0.2874	0.7126
*Delonix regia*	0.0838	0.1928	1.0000	0.7202	0.5459	0.4541
*Euphorbia pulcherrima*	0.0697	0.0759	0.0099	0.8614	0.3355	0.6645
*Impatiens walleriana*	0.0069	0.6244	1.0000	0.8613	0.0561	0.9439
*Ipomoea nil*	0.0462	0.3417	1.0000	0.3775	0.9635	0.0365
*Lantana camara*	0.0478	0.1908	1.0000	0.8856	0.5750	0.4250
*Oxalis debilis*	0.0506	0.2727	1.0000	0.8321	0.2138	0.7862
*Vachellia farnesiana*	0.1393	0.0919	0.0495	0.5565	0.9982	0.0018

**Table 2:** Parameters for the regression analysis.

IOPC	R^2^	Regression Coefficient (b, 1 × 10^6^ km^2^/°C)	*p*
*B. glabra*	0.93	26.72	4.17 × 10^−10^
*C. roseus*	0.94	15.23	2.48 × 10^−10^
*D. regia*	0.94	13.13	1.55 × 10^−10^
*E. pulcherrima*	0.86	27.36	9.58 × 10^−8^
*I. walleriana*	0.86	8.93	9.03 × 10^−8^
*I. nil*	0.85	14.78	1.73 × 10^−7^
*L. camara*	0.94	22.59	1.29 × 10^−10^
*O. debilis*	0.66	9.40	8.28 × 10^−5^
*V. farnesiana*	0.93	4.47	4.46 × 10^−10^

**Table 3:** Temperature increases and potentially suitable areas for IOPCs under future climate scenarios.

Climate Change Scenario	Average Temperature Rise (°C)	Potential Distribution Area Change Rate (%)								
		*B. glabra*	*C. roseus*	*D. regia*	*E. pulcherrima*	*I. walleriana*	*I. nil*	*L. camara*	*O. debilis*	*V. farnesiana*
SSP370_2021-2040	0.998	22.756	11.035	15.374	22.070	14.027	52.360	17.016	29.037	13.214
SSP126_2021-2040	1.248	33.781	21.494	28.044	29.847	18.645	71.936	28.743	29.439	19.882
SSP245_2021-2040	1.312	34.421	29.611	25.050	32.564	21.696	106.551	35.691	45.773	15.530
SSP585_2021-2040	1.368	49.386	42.653	36.627	56.979	22.862	145.976	52.993	53.341	18.765
SSP126_2061-2080	1.727	25.630	18.199	21.041	31.758	12.817	67.591	24.468	36.144	13.403
SSP126_2081-2100	1.818	48.713	32.012	37.807	53.283	21.797	105.789	45.734	47.129	23.651
SSP126_2041-2060	1.881	67.324	45.796	55.602	75.720	26.964	127.359	66.612	45.840	23.004
SSP370_2041-2060	1.979	73.158	57.963	66.780	97.248	32.609	126.906	85.187	53.528	30.352
SSP245_2041-2060	2.025	20.031	9.891	12.122	18.316	11.466	57.776	16.173	28.307	10.759
SSP585_2041-2060	2.583	50.871	34.051	47.152	42.442	19.524	115.074	46.262	37.853	21.227
SSP245_2061-2080	2.616	76.294	65.691	78.227	98.149	30.499	188.221	91.744	52.342	32.540
SSP245_2081-2100	2.967	80.989	71.255	75.678	96.870	32.924	165.934	98.889	54.837	32.297
SSP370_2061-2080	3.172	30.038	23.812	21.533	34.333	17.277	86.086	30.551	44.966	13.765
SSP585_2061-2080	3.606	59.222	43.127	43.947	59.958	23.398	121.233	62.470	46.661	23.145
SSP370_2081-2100	3.991	78.963	76.965	82.498	81.238	29.265	229.735	105.311	69.339	31.075
SSP585_2081-2100	4.691	87.784	82.332	93.810	95.650	31.096	241.512	111.736	55.320	35.777

## Data Availability

The original contributions presented in this study are included in the article/Supplementary Material. Further inquiries can be directed to the corresponding author.

## Supplementary Materials

The following supporting information can be downloaded at: https://www.mdpi.com/article/10.3390/plants14091361/s1, Table S1: Names and sources of the 17 environmental variables used for species distribution models of the nine introduced ornamental plants; Figure S1: Potential distribution area of the nine introduced ornamental plants under the current and future scenarios; Figure S2: Correlation analysis of 17 environmental variables; Figure S3: Accuracy assessment of single models for the nine IOPCs; Figure S4: Accuracy assessment of ensemble models for the nine IOPCs; Figure S5: Environmental background of the native ranges of the nine introduced ornamental plants; Figure S6: Environmental background of the introduced range in China for the nine introduced ornamental plants; Figure S7: Niche similarity test for niche comparison of the nine introduced ornamental plants; Figure S8: Niche equivalency test for niche comparison of the nine introduced ornamental plants; Figure S9: *Bougainvillea glabra* future potential distribution under climate change scenarios; Figure S10: *Catharanthus roseus* future potential distribution under climate change scenarios; Figure S11: *Delonix regia* future potential distribution under climate change scenarios; Figure S12: *Euphorbia pulcherrima* future potential distribution under climate change scenarios; Figure S13: *Impatiens walleriana* future potential distribution under climate change scenarios; Figure S14: *Ipomoea nil* future potential distribution under climate change scenarios; Figure S15: *Lantana camara* future potential distribution under climate change scenarios; Figure S16: *Oxalis debilis* future potential distribution under climate change scenarios; Figure S17: *Vachellia farnesiana* future potential distribution under climate change scenarios.

## Abbreviations

The following abbreviations are used in this manuscript:

IOPC	Introduced ornamental plants in China
SDM	Species distribution model
PCA	Principal component analysis
