# Supplementary material for: The Ecological Risks and Invasive Potential of Introduced Ornamental Plants in China
Source: Plants (Basel). 2025 Apr 30;14(9):1361. doi: 10.3390/plants14091361 (PMC12074367; doi:10.3390/plants14091361)
Supplement: Supplementary file 1 [file plants-14-01361-s001.zip › Supplementary Table.pdf]

Table S1: Names and sources of the 17 environmental variables used for species distribution models of the nine introduced ornamental plants

Classification and data source	Variables
Bioclimatic variables<br>Data source: WorldClim;<br>(http://www.worldclim.org/)	bio2: Mean Diurnal Range<br>bio3: Isothermality<br>bio6: Minimum Temperature of the Coldest Month<br>bio10: Mean Temperature of the Warmest Quarter<br>bio12: Annual Precipitation<br>bio15: Precipitation Seasonality<br>bio19: Precipitation of the Coldest Quarter
Topographic variable<br>,Data source: WorldClim	elev: Elevation
Soil variables<br>Data source: Harmonized World Soil Database;(https://gaez.fao.org/pages/hwsd)	D1_ALUM_SAT: Aluminum Saturation<br>D1_ESP: Exchangeable Sodium Percentage<br>D2_BSAT: Base Saturation<br>D2_CEC_EFF: Effective Cation Exchange C apacity<br>D2_COARSE: Coarse Fragments<br>D2_PH_WATER: Soil pH in Water
Ultraviolet (UV) radiation variables<br>Data source: Global UV-B Radiation Dataset;(https://www.ufz.de/gluv/)	UVB2: UV-B_Seasonality<br>UVB4: Mean_UV-B_of_Lowest_Month<br>UVB5: Sum_of_UV-B_Radiation_of_Highest _Quarter
