# Supplementary figures and images for: The Ecological Risks and Invasive Potential of Introduced Ornamental Plants in China
Source: Plants (Basel). 2025 Apr 30;14(9):1361. doi: 10.3390/plants14091361 (PMC12074367; doi:10.3390/plants14091361)

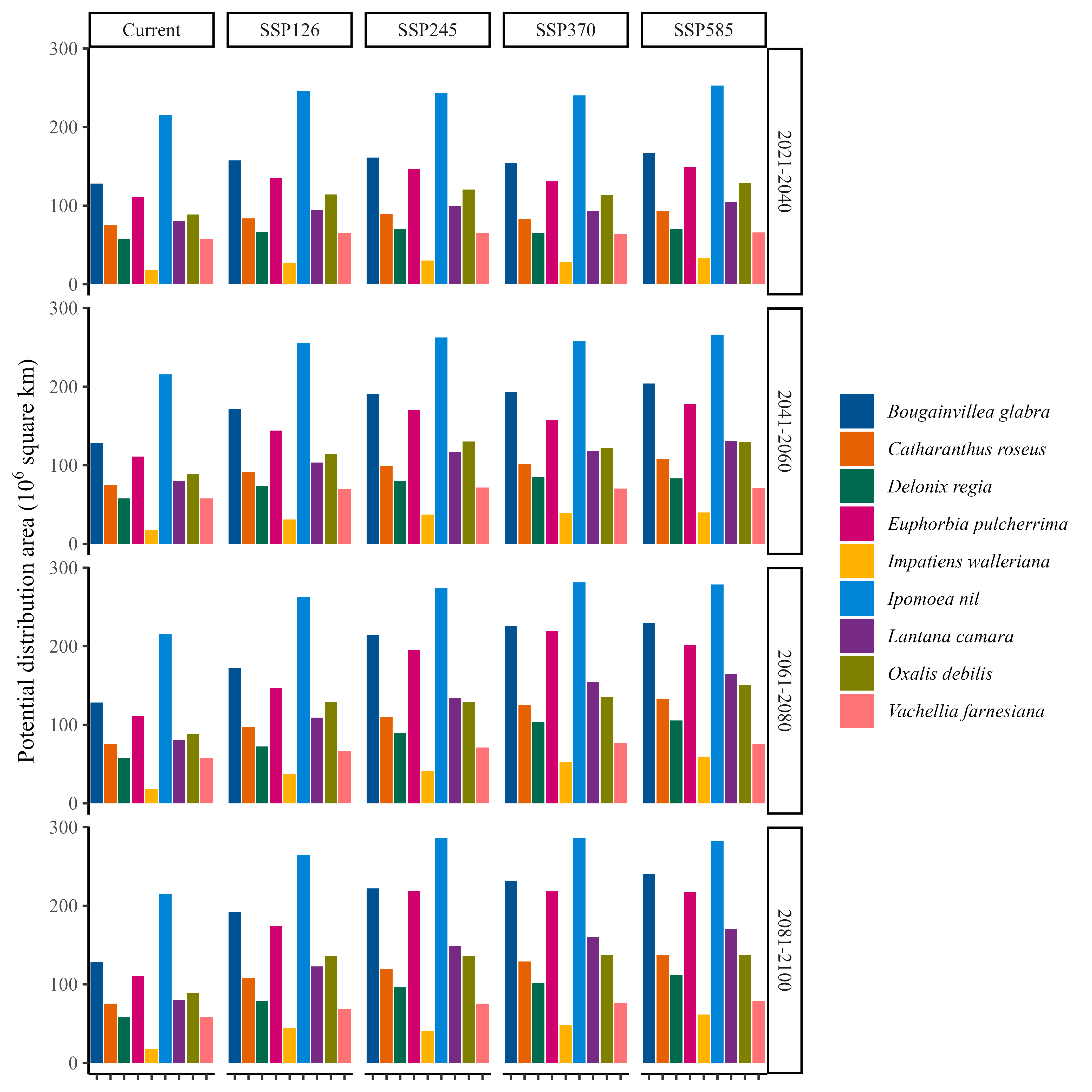

Supplement: Supplementary file 1 [file plants-14-01361-s001.zip › Supplementary Figures/Figure S1.tif]

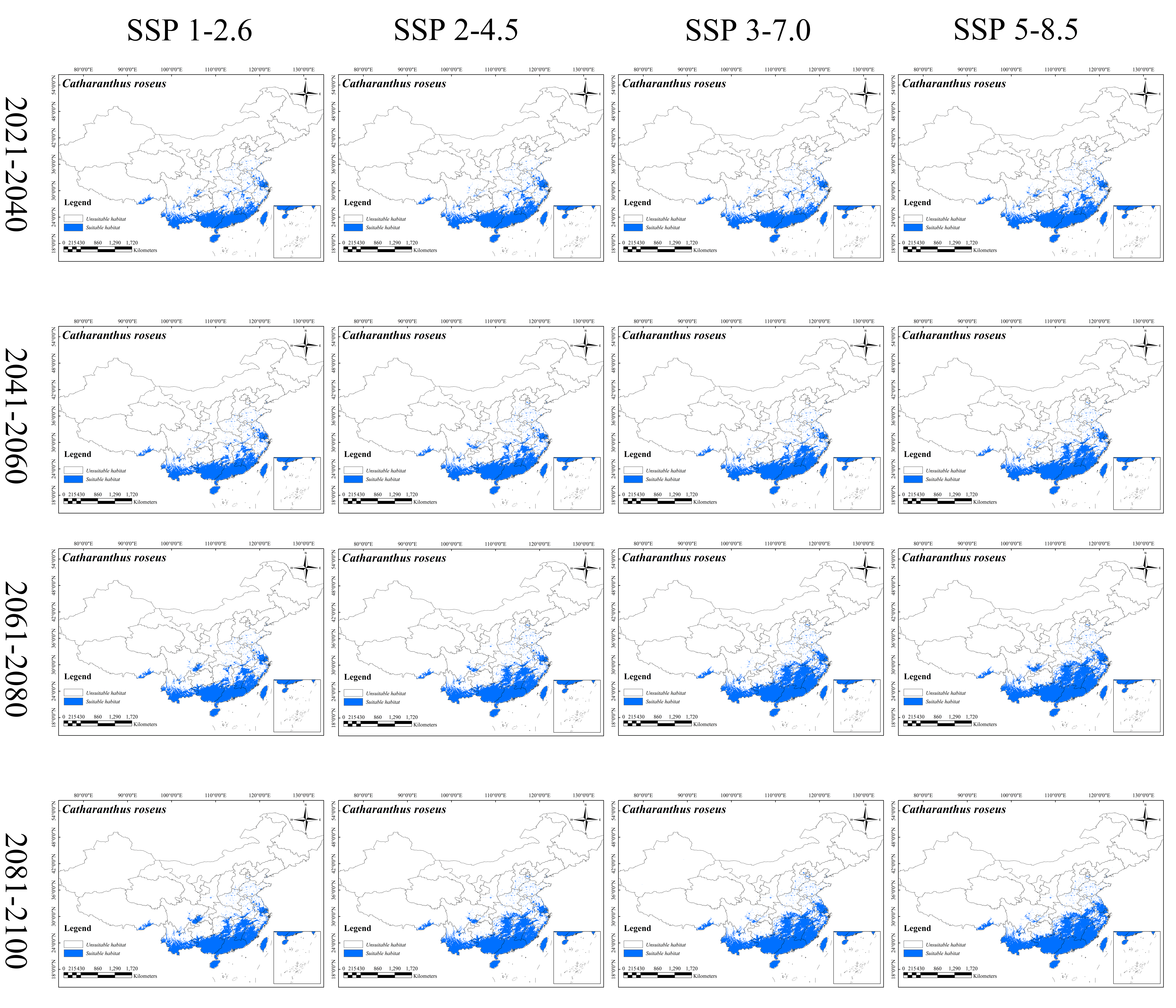

Supplement: Supplementary file 1 [file plants-14-01361-s001.zip › Supplementary Figures/Figure S10.tif]

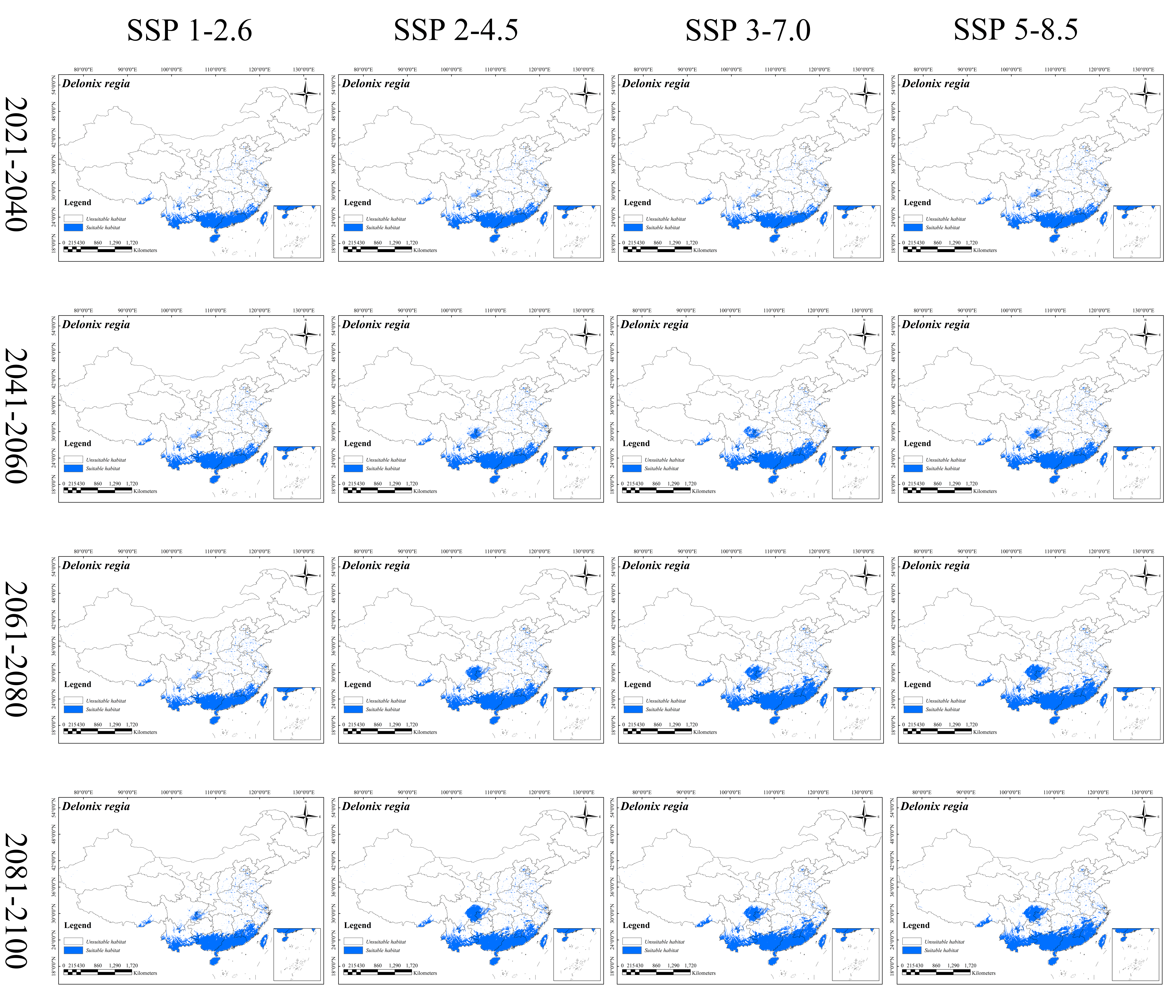

Supplement: Supplementary file 1 [file plants-14-01361-s001.zip › Supplementary Figures/Figure S11.tif]

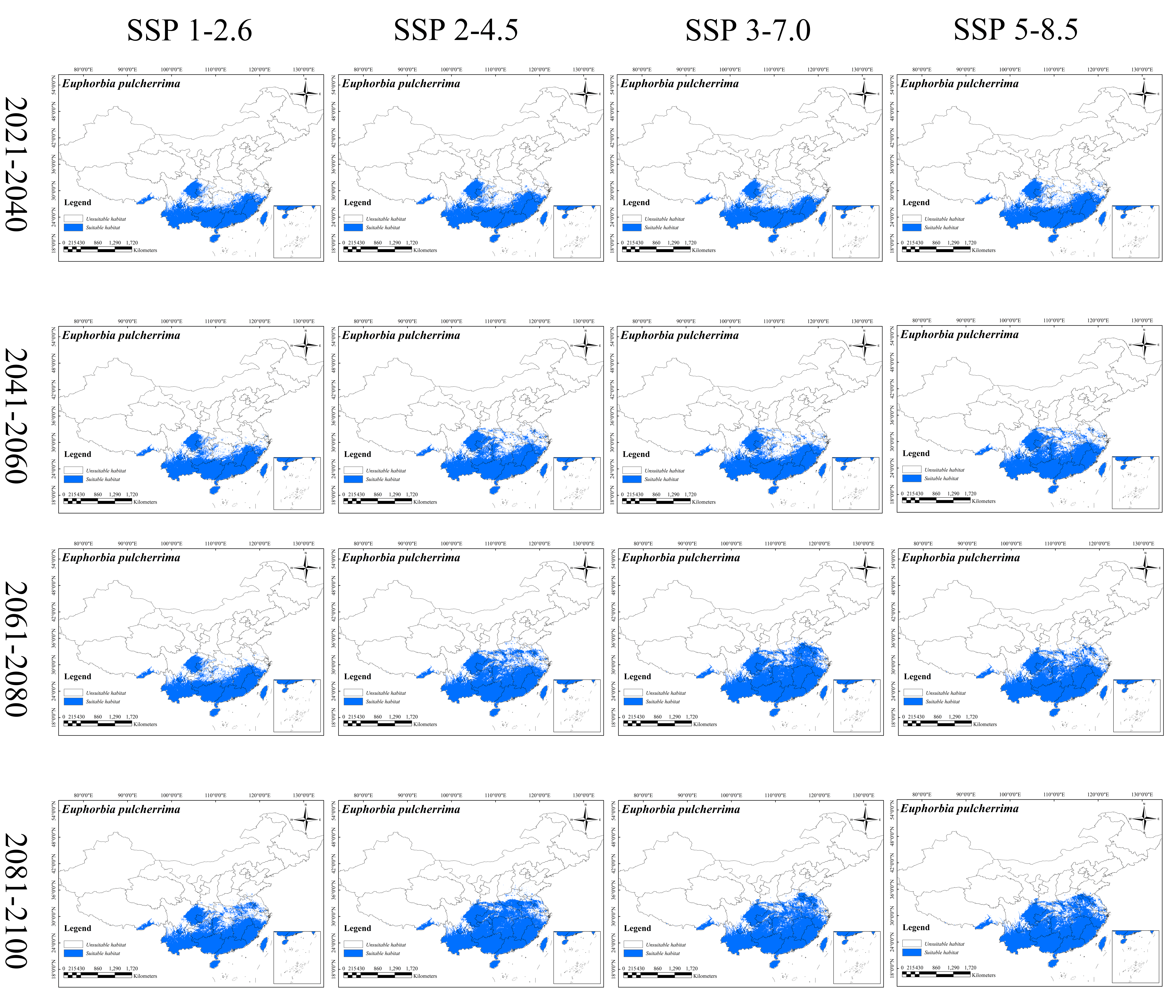

Supplement: Supplementary file 1 [file plants-14-01361-s001.zip › Supplementary Figures/Figure S12.tif]

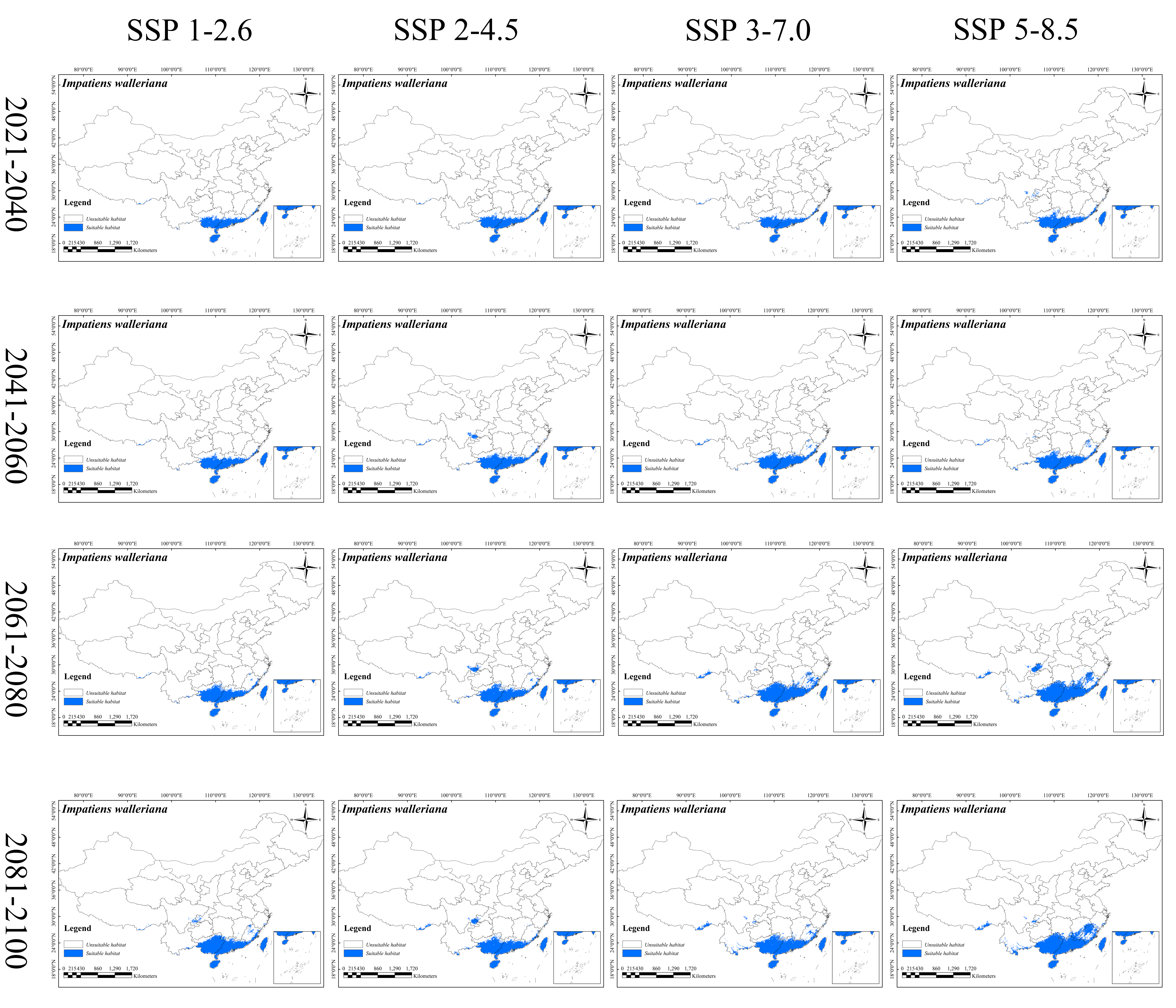

Supplement: Supplementary file 1 [file plants-14-01361-s001.zip › Supplementary Figures/Figure S13.tif]

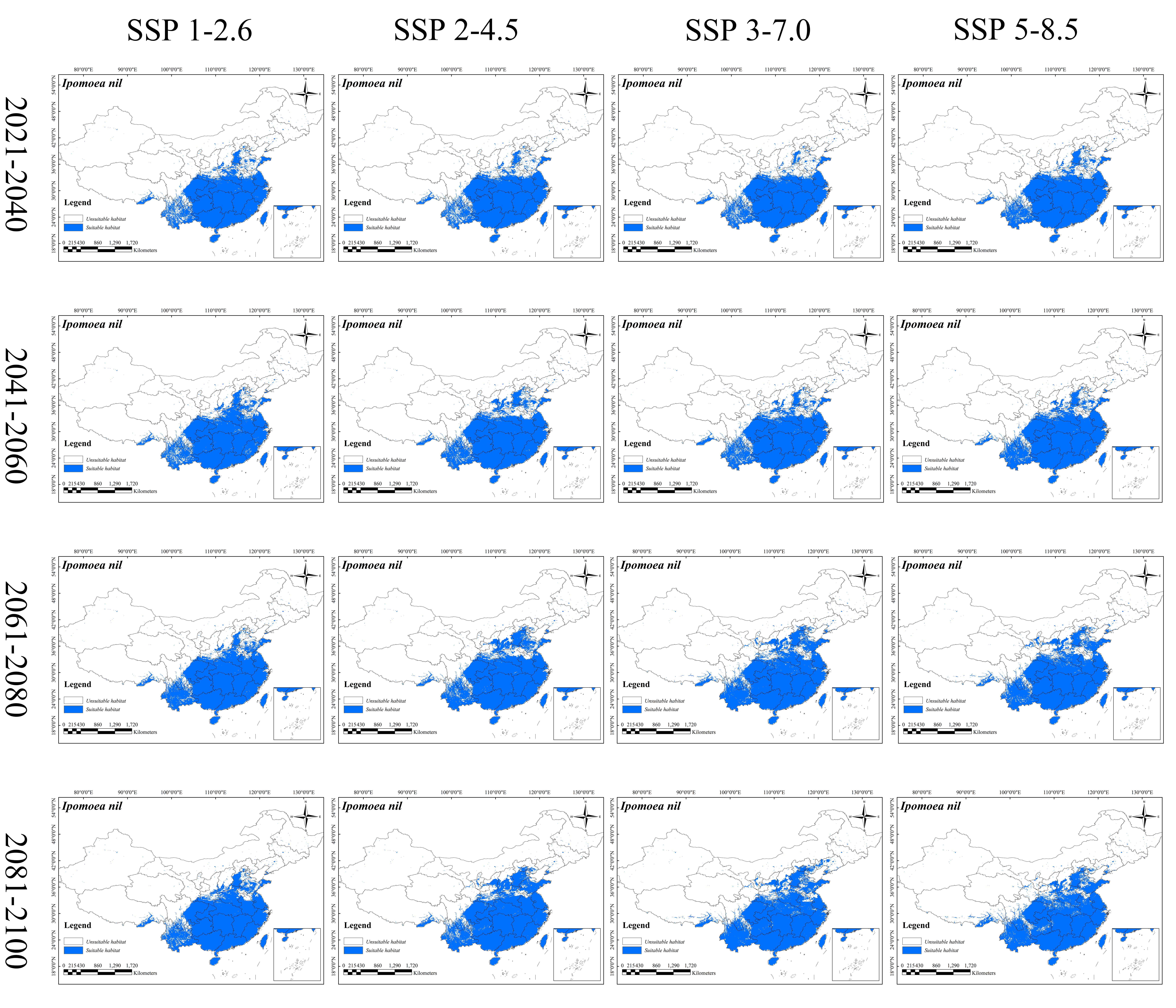

Supplement: Supplementary file 1 [file plants-14-01361-s001.zip › Supplementary Figures/Figure S14.tif]

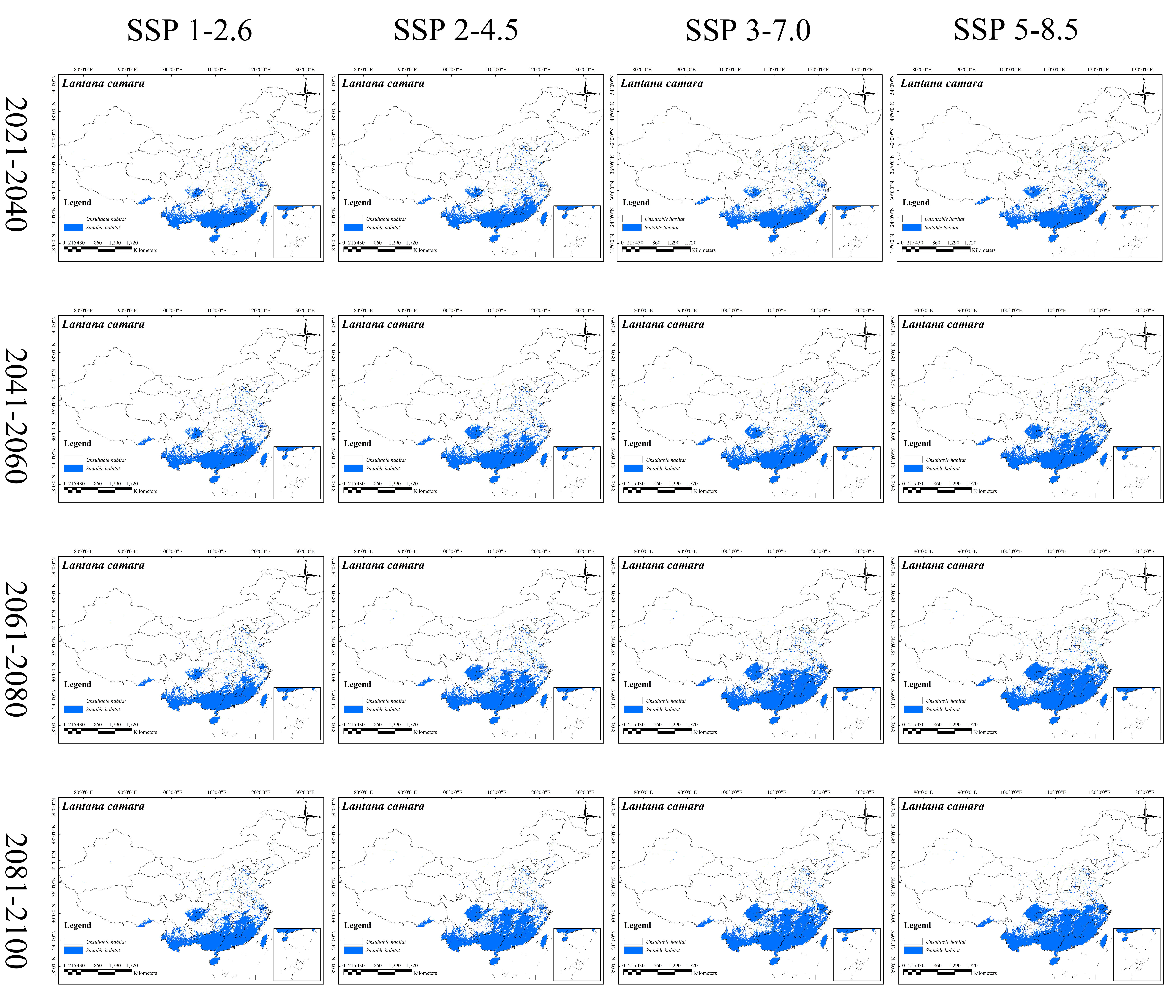

Supplement: Supplementary file 1 [file plants-14-01361-s001.zip › Supplementary Figures/Figure S15.tif]

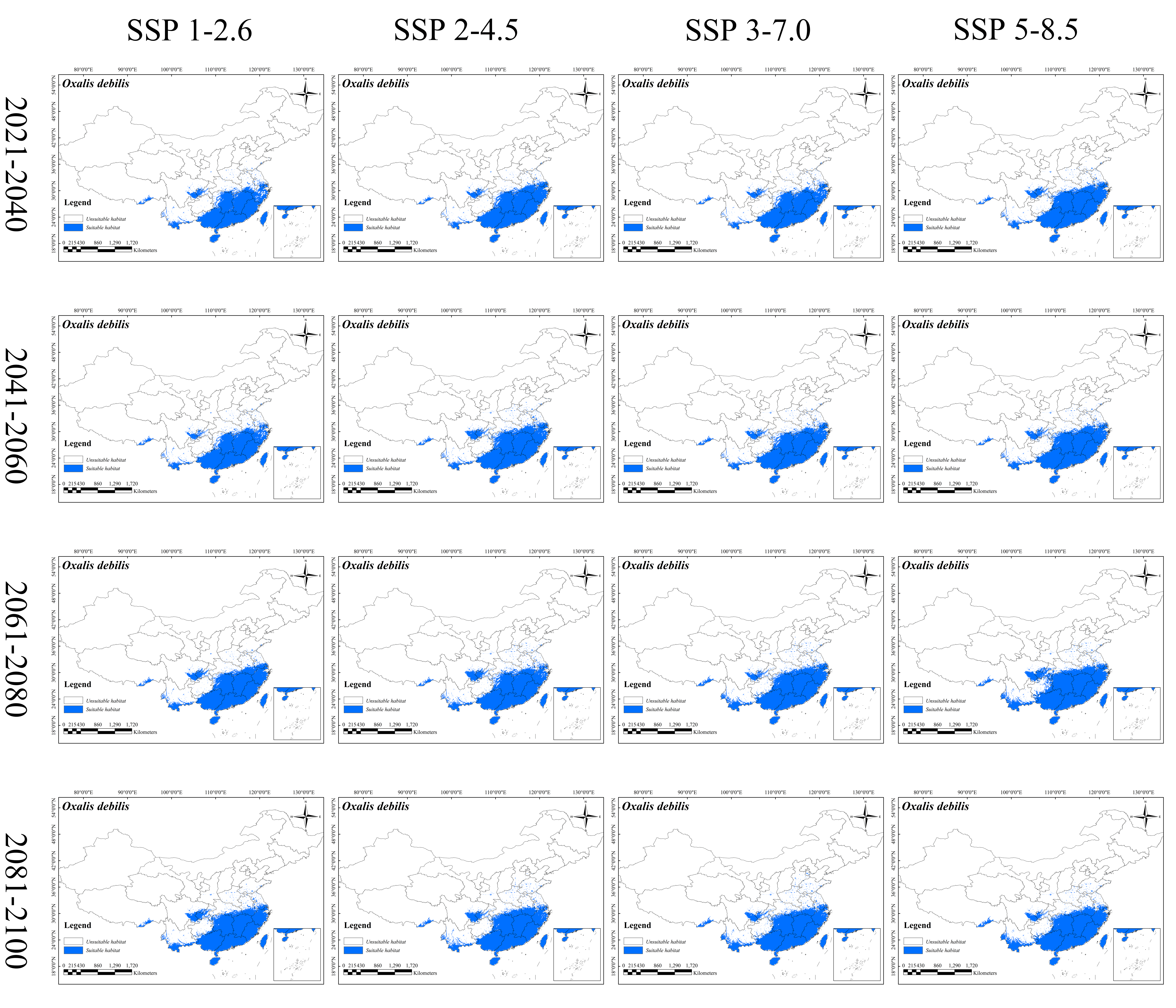

Supplement: Supplementary file 1 [file plants-14-01361-s001.zip › Supplementary Figures/Figure S16.tif]

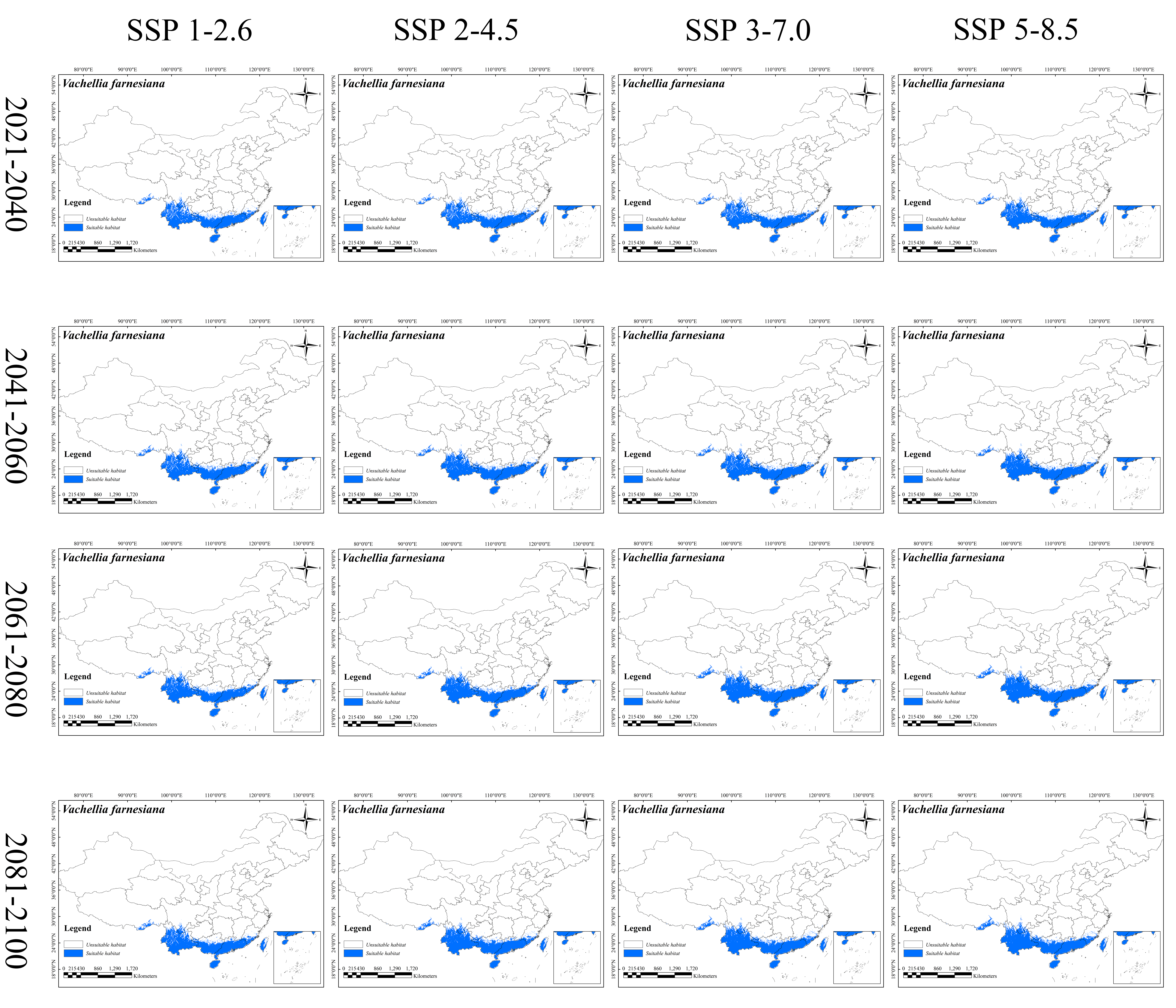

Supplement: Supplementary file 1 [file plants-14-01361-s001.zip › Supplementary Figures/Figure S17.tif]

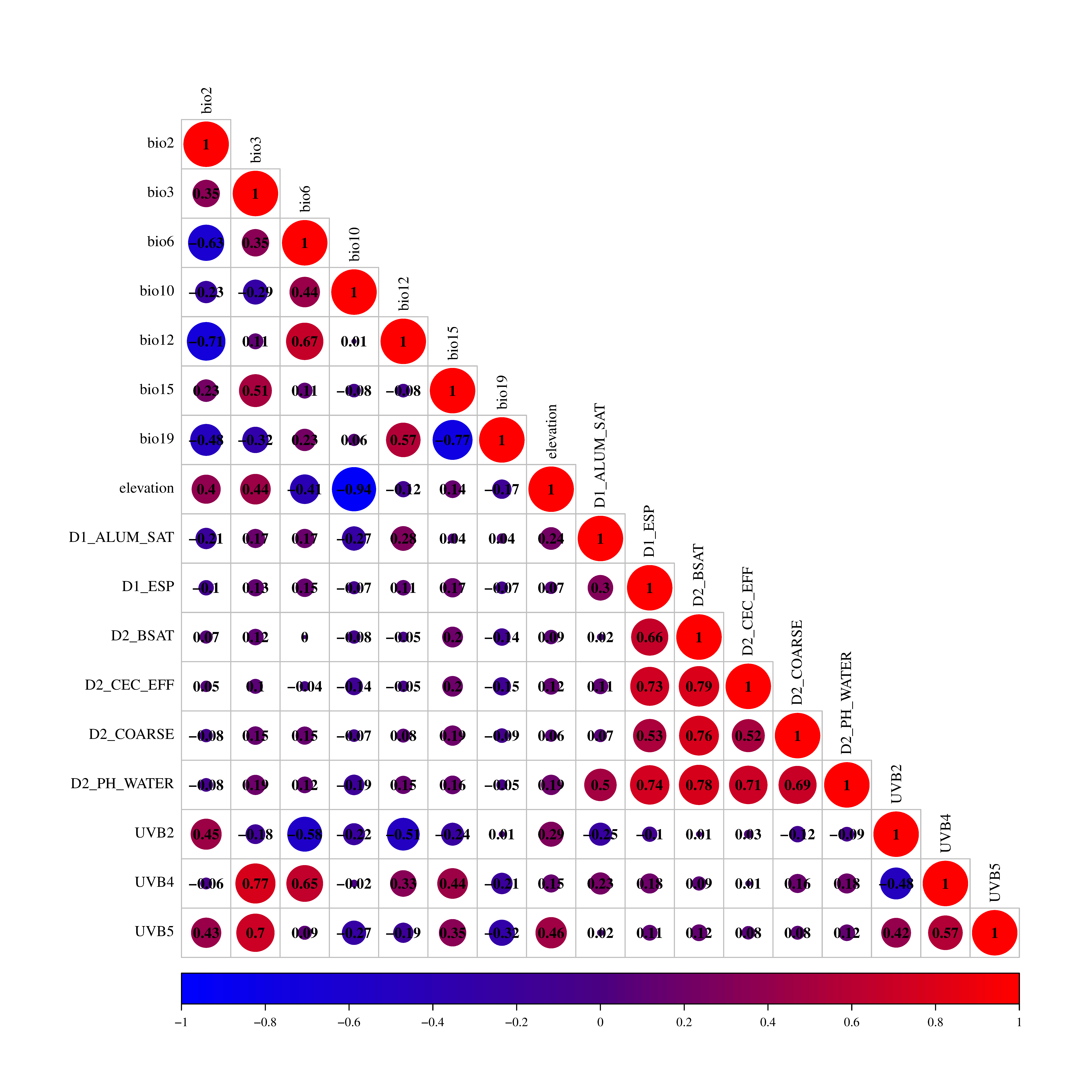

Supplement: Supplementary file 1 [file plants-14-01361-s001.zip › Supplementary Figures/Figure S2.tif]

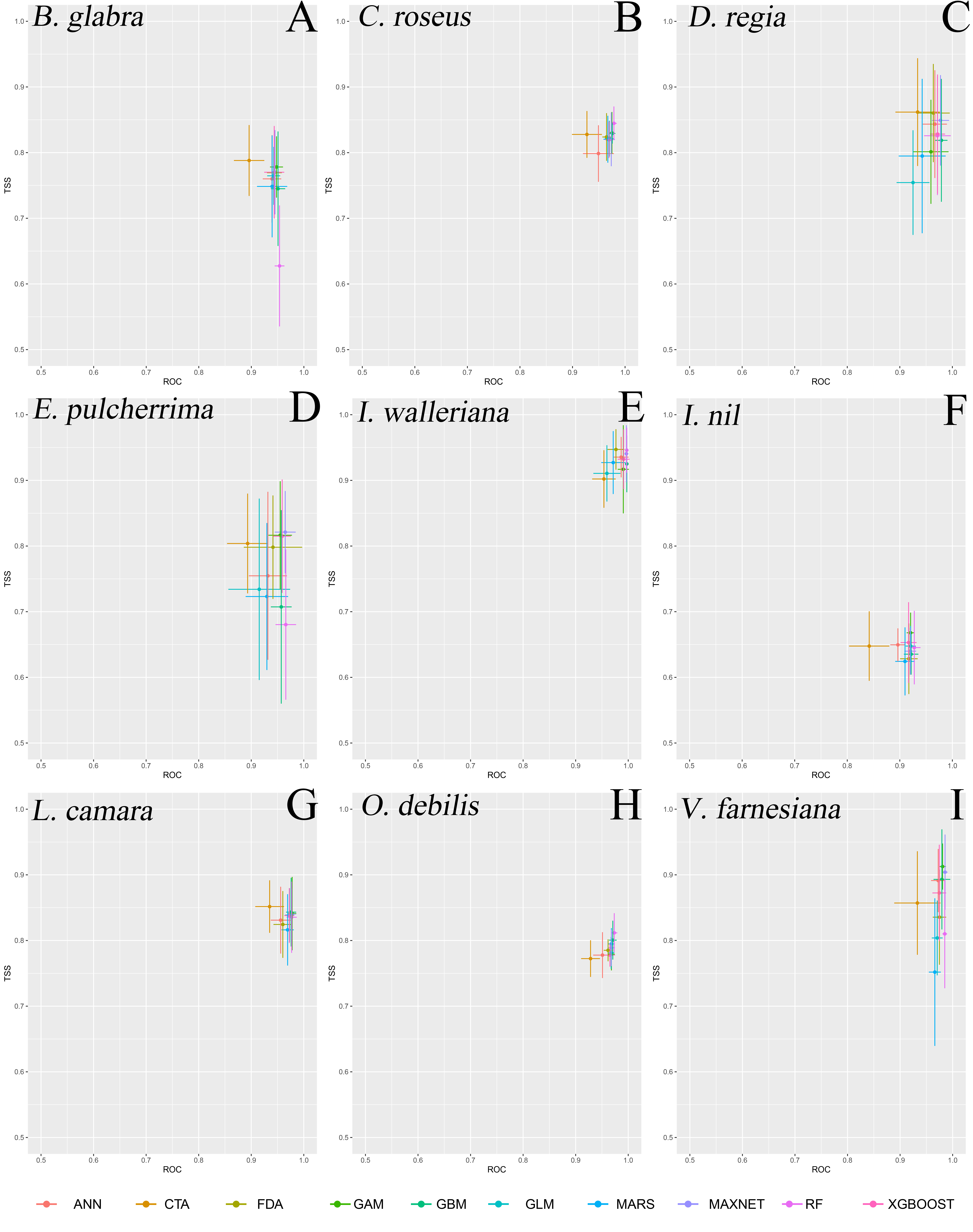

Supplement: Supplementary file 1 [file plants-14-01361-s001.zip › Supplementary Figures/Figure S3.jpg]

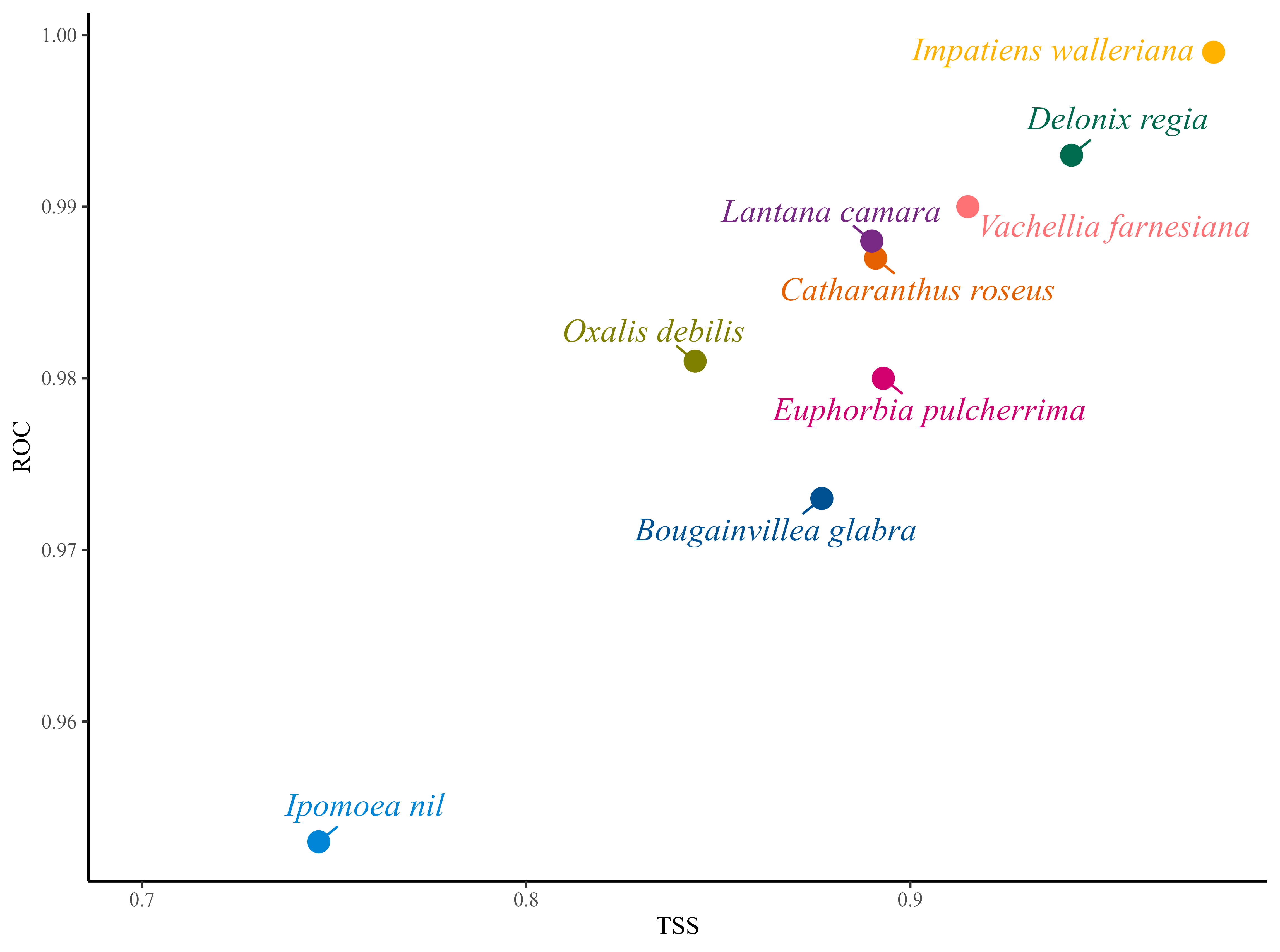

Supplement: Supplementary file 1 [file plants-14-01361-s001.zip › Supplementary Figures/Figure S4.tif]

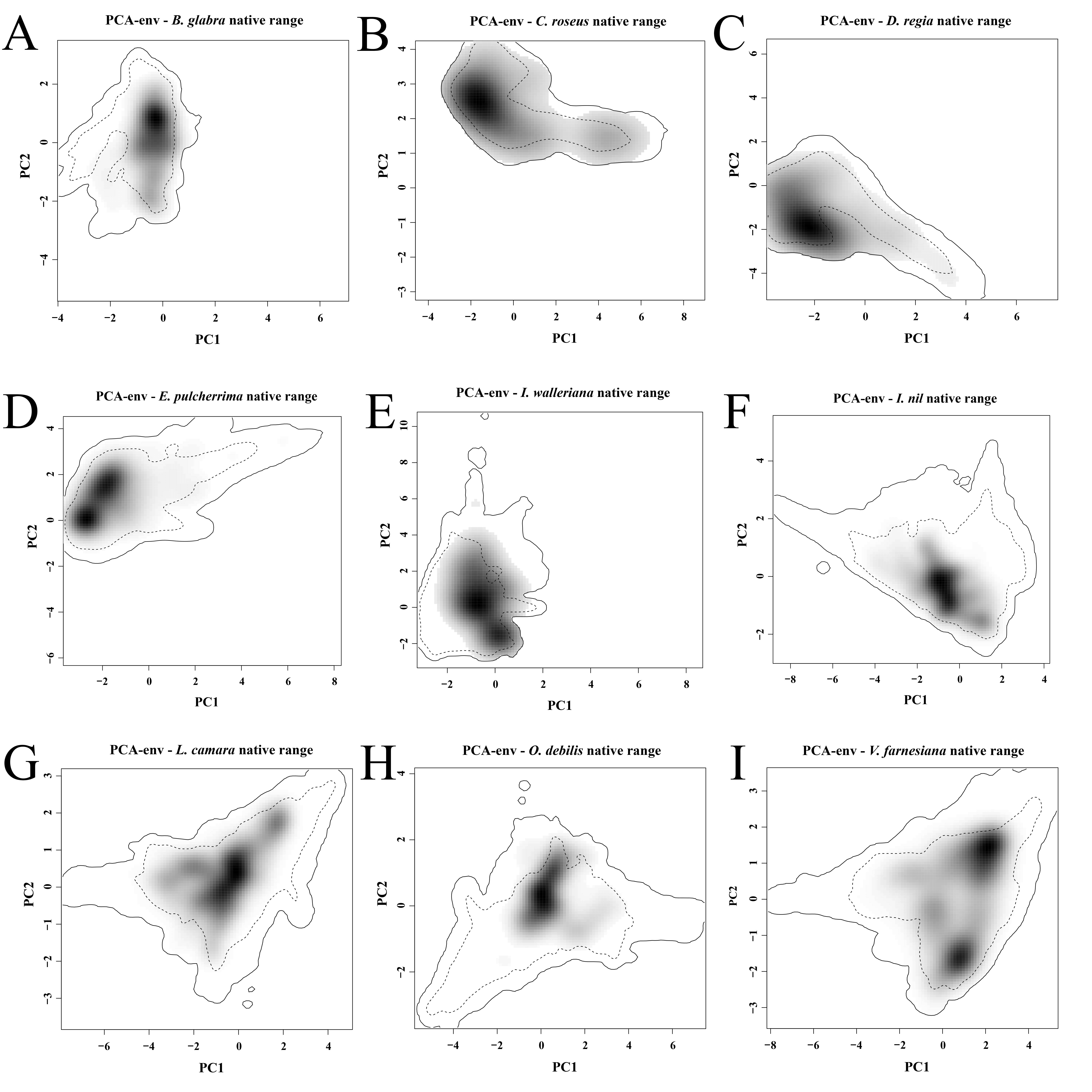

Supplement: Supplementary file 1 [file plants-14-01361-s001.zip › Supplementary Figures/Figure S5.tif]

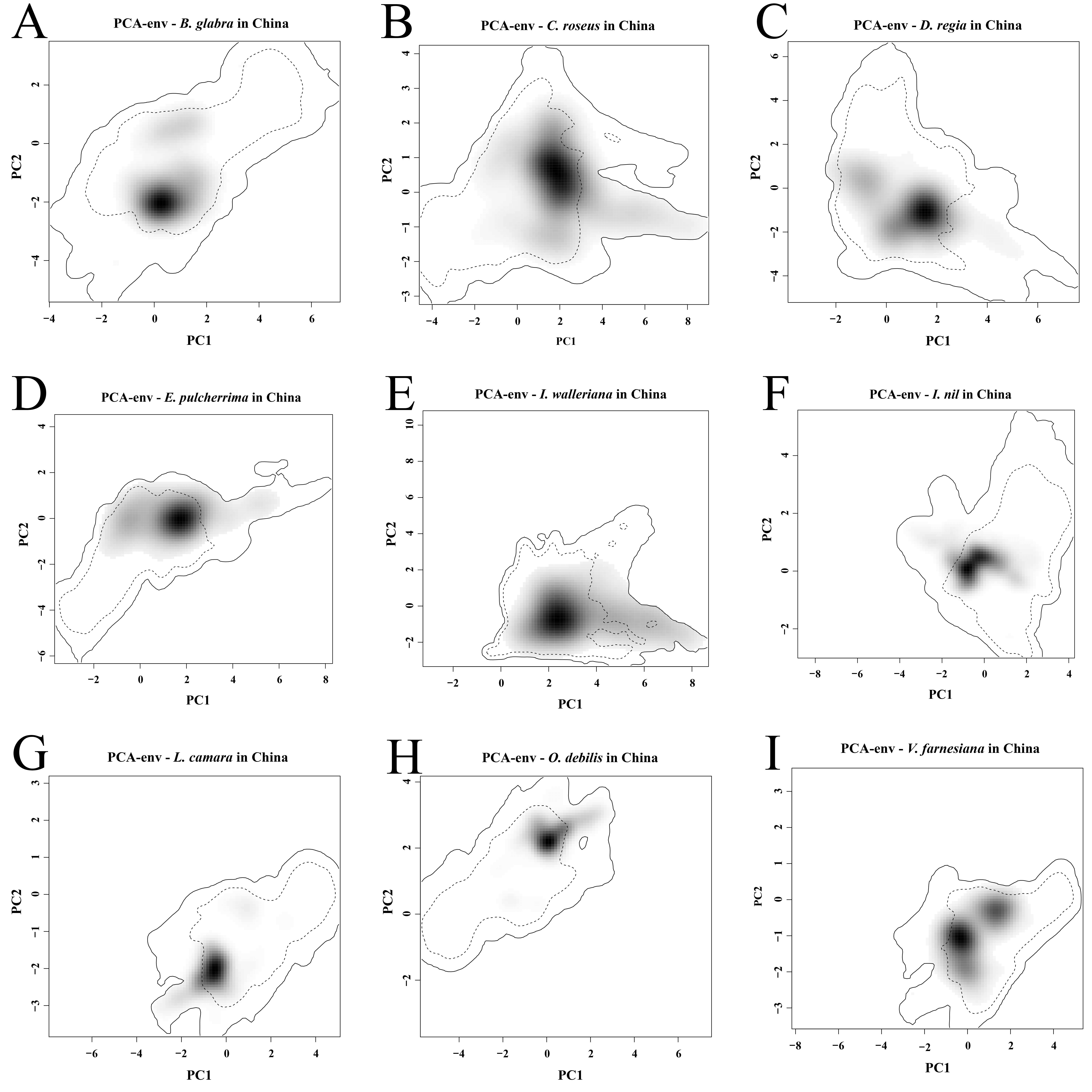

Supplement: Supplementary file 1 [file plants-14-01361-s001.zip › Supplementary Figures/Figure S6.tif]

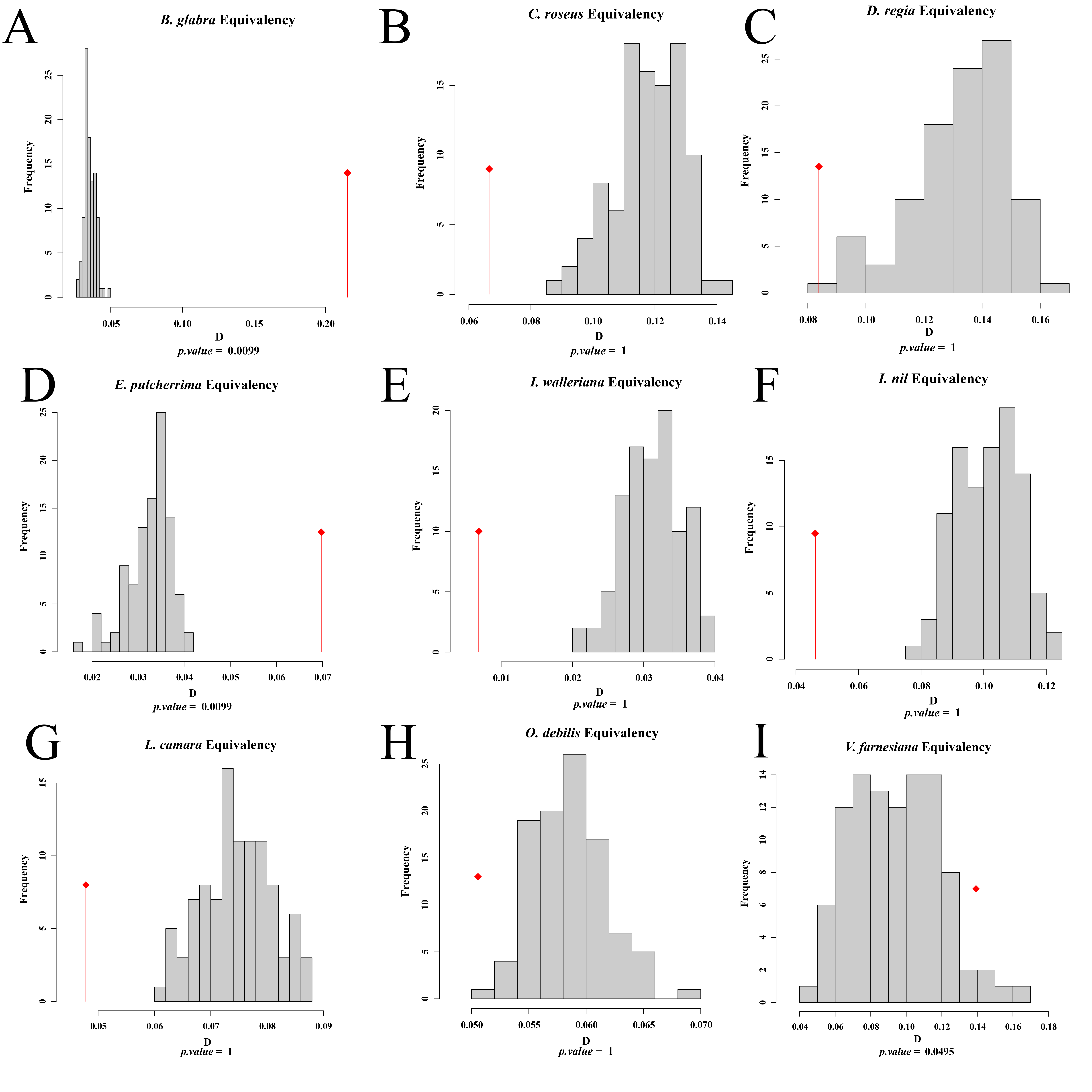

Supplement: Supplementary file 1 [file plants-14-01361-s001.zip › Supplementary Figures/Figure S7.tif]

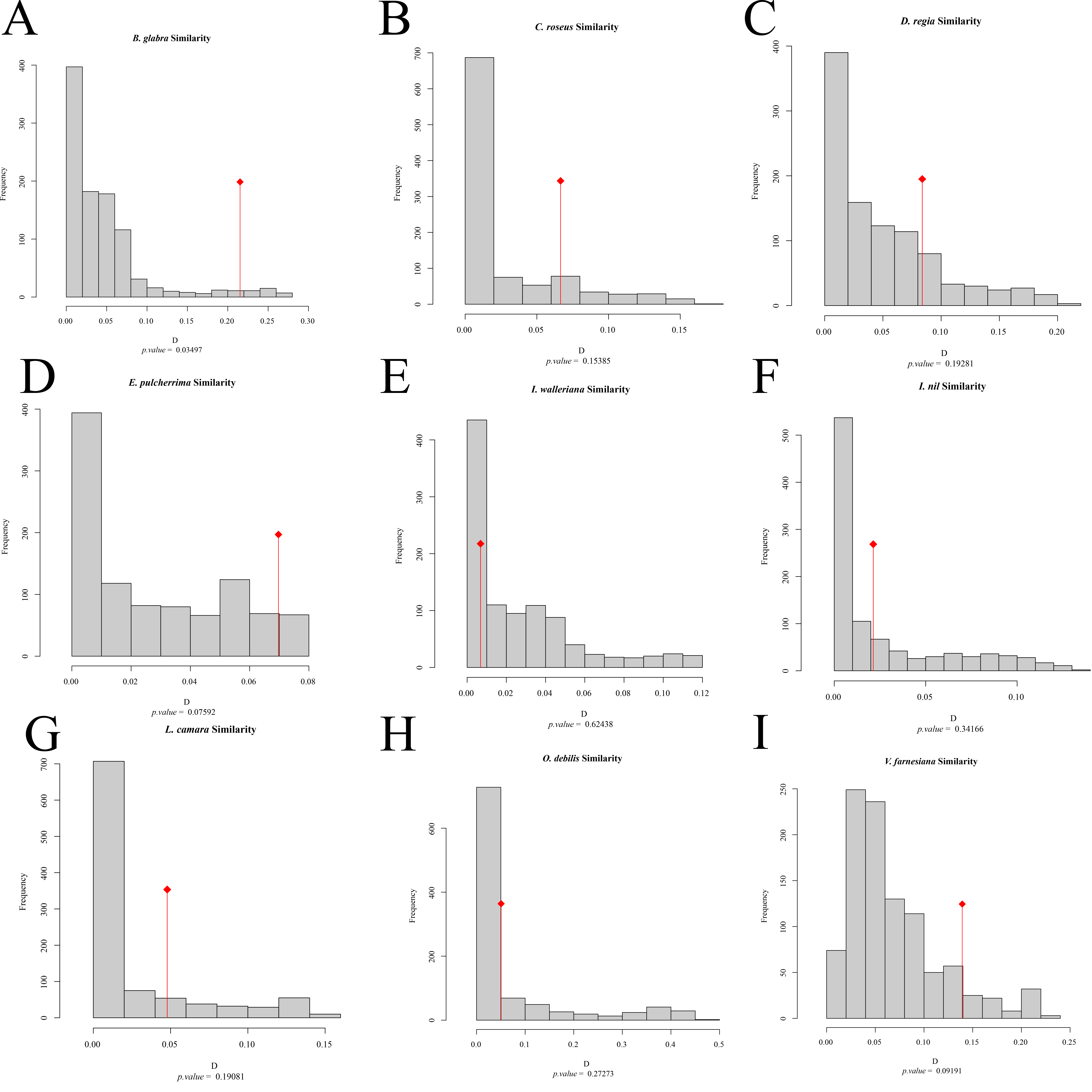

Supplement: Supplementary file 1 [file plants-14-01361-s001.zip › Supplementary Figures/Figure S8.tif]

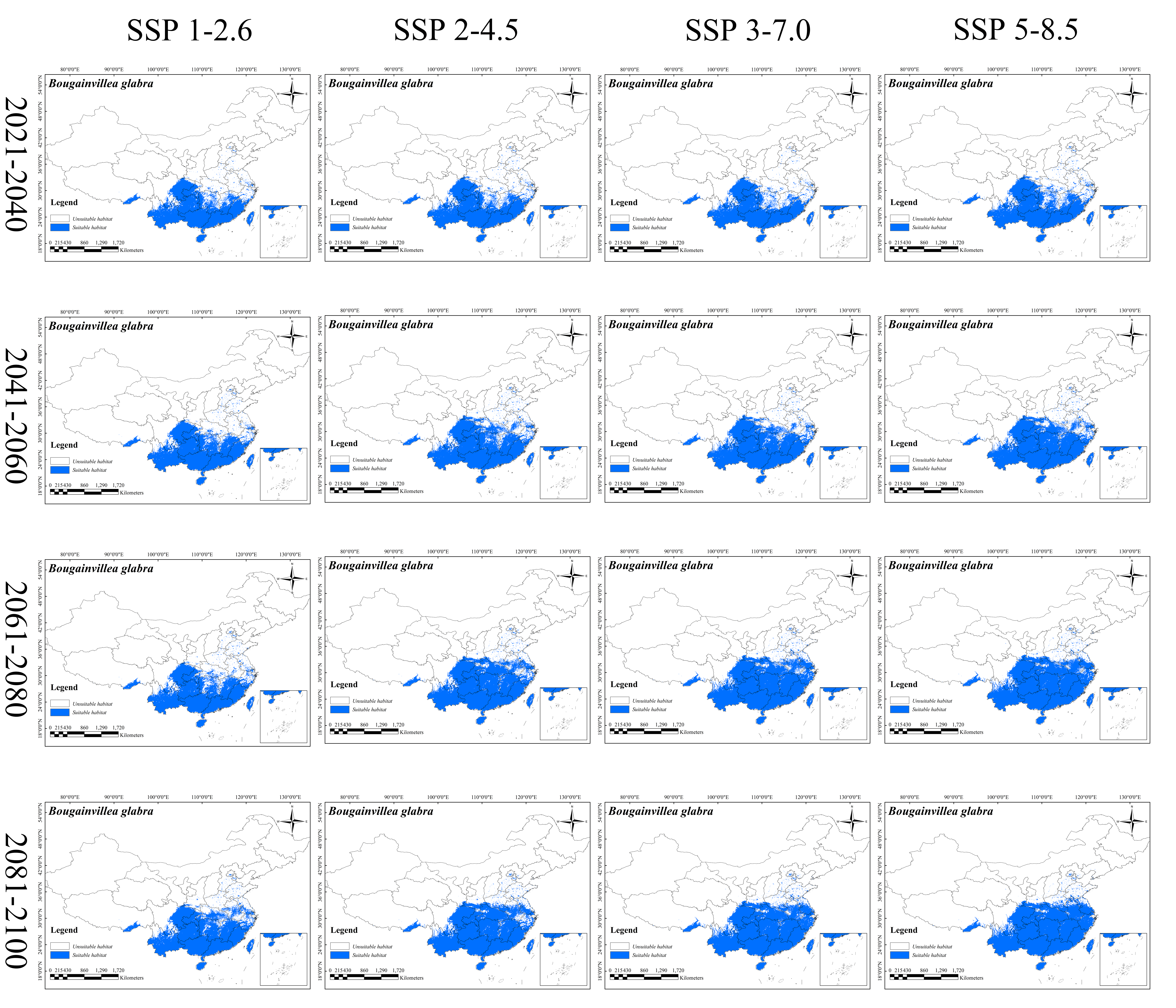

Supplement: Supplementary file 1 [file plants-14-01361-s001.zip › Supplementary Figures/Figure S9.tif]
